# From Engel's Bio-Psycho-Social model to the personalized health determinants model: a comprehensive framework and illustrative operationalization for precision health

**DOI:** 10.3389/fdgth.2026.1763691

**Published:** 2026-06-10

**Authors:** Nazanin Abbaspour, Mory Ghomshei, Ario Saeid Vaghefi, Karim Abbaspour

**Affiliations:** 1 Family Health Centers of San Diego, San Diego, CA, United States; 2 NutraLumina Integrative & Functional Nutrition, San Diego, CA, USA; 3 Tecto Energy Inc, Vancouver, BC, Canada; 4 Carnotech Energy Inc, Vancouver, BC, Canada; 5 Department of Geography, University of Zurich, Zurich, Switzerland; 6 WMO, Geneva, Switzerland; 7 2w2e Consulting, GmbH, Dübendorf, Switzerland

**Keywords:** bayesian networks, bio-psycho-social model, digital health tools, health equity, obesity risk prediction, personalized health determinants, precision health, rule-Based scoring

## Abstract

Engel's Bio-Psycho-Social (BPS) model (1977) reframed healthcare by integrating biological, psychological, and social perspectives. Despite its influence, the model has been criticized for insufficient specificity in domains critical to precision health, including nutrition, lifestyle, socioeconomic, environmental, and structural factors. To address these limitations, we propose the Personalized Health Determinants Model (PHDm), a comprehensive nine-dimension framework, Biological, Psychological, Social, Cultural, Environmental, Economic, Political, Spiritual, and Lifestyle, synthesized from Engel's BPS model, the WHO Commission on Social Determinants of Health, CDC frameworks, and related literature. Section 2.0 provides a detailed evidence-based rationale for the dimensions and their relationship to prior frameworks. The PHDm organizes each dimension hierarchically into categories, sub-categories, and measurable health elements (theoretical maximum −72,000), with only tailored, clinically relevant subsets applied in practice. Using obesity as a case study, we illustrate operationalization through a Bayesian Network and a complementary rule-based scoring system. Four exemplar factors, insulin sensitivity, dietary fiber, caloric intake, and activity frequency, are mapped into both models, enabling individualized obesity risk quantification and intervention simulation. Preliminary evaluation on NHANES data yielded −85% predictive accuracy for the Bayesian Network and −80% concordance for the rule-based system. This manuscript presents the PHDm as a conceptual framework with illustrative operationalization. While the Bayesian Network and rule-based approaches demonstrate promising preliminary performance on NHANES data, the current model, based on four simplified factors and binary thresholds, remains a proof-of-concept prototype and is not yet a clinically validated decision-support tool. Further development, external validation, and integration into electronic health records will be required before clinical deployment.

## Highlights

1. The PHDm offers a comprehensive nine-dimension framework that expands Engel's BPS model.
2. It provides an illustrative operationalization using Bayesian Networks and rule-based scoring on an obesity case study.
3. The current prototype shows promising preliminary performance but remains a proof-of-concept.
4. Significant further validation across diverse populations and clinical settings is required.
5. We invite interdisciplinary collaboration to advance this testable framework for precision health.

## 1 Introduction

How can clinicians integrate thousands of interconnected health determinants into daily practice without overwhelming complexity? Health is a complex, multidimensional phenomenon shaped by the dynamic interaction of biological, psychological, social, and economic factors. In 1977, George Engel's Bio-Psycho-Social (BPS) model (1) provided a groundbreaking alternative to the reductionist biomedical paradigm by emphasizing a holistic view of health and disease. Despite its transformative impact, the BPS model has been criticized for lacking specificity in domains critical for effective healthcare planning, such as nutrition, lifestyle, socioeconomic conditions, and environmental exposures (2, 3). For example, nutrition plays a central role in the management of chronic diseases such as diabetes, which affects over 850 million adults worldwide (4), yet the BPS framework offers little guidance for integrating nutritional science into clinical decision-making.

Subsequent efforts to expand the BPS model have sought to address these gaps, yet many expansions remain largely theoretical, with limited integration of computational methods for practical application (5–7). For instance, the Biobehavioral Family Model extends BPS by highlighting reciprocal influences within family systems on health outcomes, particularly in chronic illness management (7). Similarly, the biopsychosociotechnical model integrates sociotechnical systems theory to account for technology's role in health behaviors, as seen in digital interventions for mental health (6). Recent calls for revival underscore the model's enduring relevance but stress the urgency of making it more dynamic and testable, moving beyond conceptual breadth to quantifiable, context-specific applications (5, 8, 9).

To address these limitations, we propose the Personalized Health Determinants Model (PHDm), which expands Engel's framework into nine interrelated dimensions, Biological, Psychological, Social, Cultural, Environmental, Economic, Political, Spiritual, and Lifestyle, while providing a clear pathway for operationalization in contemporary healthcare.

Section 2.0 presents a detailed rationale for the selection of these dimensions, their empirical justification, and how the PHDm builds upon and extends established frameworks. Section 3 describes the PHDm's hierarchical classification system and illustrates its application through an obesity case study focused on the Biological and Lifestyle dimensions. Subsequent sections demonstrate illustrative operationalization via Bayesian Networks (Section 4) and a complementary rule-based scoring system (Section 7), followed by clinical application, limitations, and future directions.

Recent advances in artificial intelligence (AI) and machine learning make such operationalization feasible, particularly for biopsychosocial-inspired frameworks. High-dimensional datasets spanning genetic profiles, behavioral patterns, electronic health records, environmental exposures, and social determinants can now be analyzed to quantify the impact of individual factors, explore their interactions, and simulate intervention scenarios (10, 11). For example, AI applications in psychiatry have leveraged machine learning for diagnostic precision in BPS-aligned models, such as predicting treatment outcomes by integrating psychological and social data with biomarkers (10, 12). Generative AI tools can also simulate holistic scenarios, such as virtual therapy sessions incorporating cultural and environmental contexts (13, 14). Yet barriers remain: resource constraints, infrastructure gaps, and the inherent complexity of holistic frameworks complicate implementation (2). Moreover, integrative models such as the BPS face challenges of scientific validation, as isolating and testing variables within interconnected systems remains difficult (15), and AI integration often overlooks equity in diverse populations (16).

This manuscript presents the PHDm as a conceptual framework with practical methodological demonstration. We provide the theoretical foundation and hierarchical structure of the model and illustrate its real-world utility through a detailed obesity case study using Bayesian Networks and rule-based scoring. While preliminary performance metrics are reported, this work is intended as a transparent proof-of-concept to encourage further development, rigorous validation across diverse populations, and interdisciplinary collaboration among researchers, clinicians, AI developers, and funders.

## 2 Rationale for the personalized health determinants model (PHDm) dimensions

The nine dimensions of the PHDm, Biological, Psychological, Social, Cultural, Environmental, Economic, Political, Spiritual, and Lifestyle, were selected through a deliberate synthesis of Engel's foundational Bio-Psycho-Social (BPS) model (1) with major established frameworks in health equity and precision health. The goal was to create a comprehensive yet practitioner-focused framework that addresses documented limitations of the original BPS model, particularly its lack of specificity regarding nutrition, lifestyle, socioeconomic conditions, environmental exposures, and structural influences (2, 17, 18).

Each dimension is supported by extensive empirical evidence demonstrating independent and interactive contributions to health outcomes across chronic conditions, including obesity, cardiovascular disease, and diabetes. In clinical application, the model is intentionally flexible: only tailored, high-relevance subsets of dimensions, categories, and elements are activated for any given patient, ensuring feasibility while preserving theoretical completeness.

### 2.1 Evidence-based justification for each dimension

- Biological: Retained as the core of the BPS model and all precision-health approaches; encompasses genetics, metabolism, and physiology (e.g., insulin sensitivity and energy metabolism; 19–21).
- Psychological: BPS cornerstone; strongly influences adherence, behavior change, and mental-health comorbidities (10, 12).
- Social: Explicitly retained from BPS and a central pillar of the WHO Commission on Social Determinants of Health (22) and Healthy People 2030 frameworks (23).
- Cultural: Recognized across Social Determinants of Health (SDoH) literature (23, 24) as shaping health beliefs, behaviors, help-seeking patterns, and disparities.
- Environmental: Highlighted by the WHO CSDH (22) and Powell-Wiley et al. (24) as a major upstream driver of obesity and cardiovascular outcomes through the built environment, pollutant exposures, and resource access.
- Economic: Core structural SDoH domain in WHO, CDC, and Powell-Wiley frameworks (22–24); directly modulates access to resources, nutrition, and healthcare.
- Political: Builds on structural determinants in WHO reports and the Political Determinants of Health framework (25); policies, governance, and power structures shape all other dimensions and health equity.
- Spiritual: Extension of the BPS model into a biopsychosocial–spiritual framework (26, 27); critical for coping, meaning-making, resilience, and holistic care in chronic illness.
- Lifestyle: Added explicitly to overcome well-documented BPS limitations in actionable behavioral domains (nutrition, physical activity, sleep); among the strongest modifiable predictors of obesity and chronic disease (28–33) and interacts dynamically with biological and SDoH factors.

## 3 Overview of the PHDm classification system

**Figure 1:**
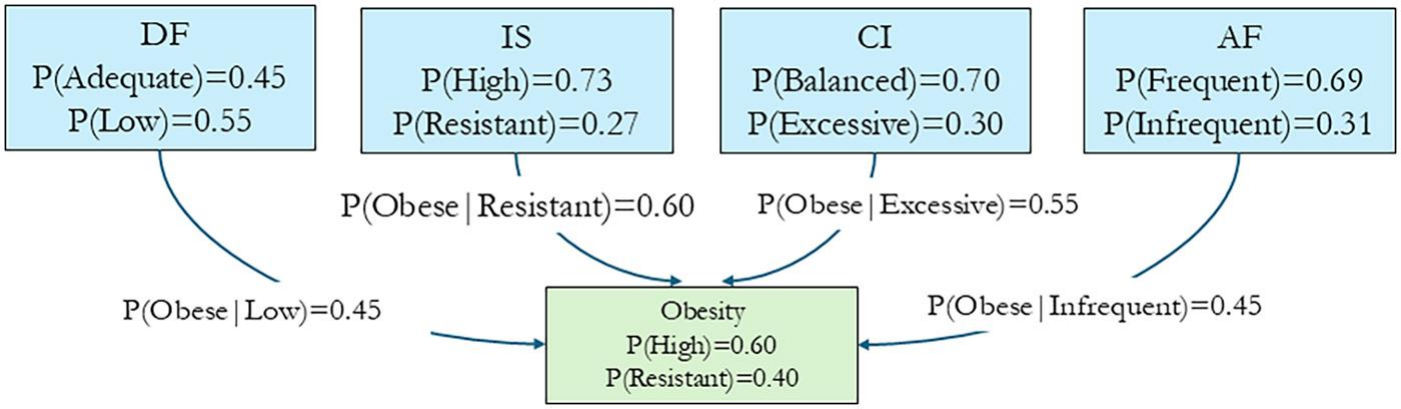
The personalized health determinants model (PHDm). This conceptual framework expands Engel's Bio-Psycho-Social model into nine interrelated dimensions—Biological, Psychological, Social, Cultural, Environmental, Economic, Political, Spiritual, and Lifestyle—to provide a more comprehensive and actionable structure for precision health. Each dimension can be hierarchically decomposed into categories, sub-categories, and measurable health elements (see Section 2.0 for detailed rationale and Supplementary Tables S1–S3 for the full taxonomy). In clinical practice, only tailored subsets of these dimensions and elements are activated for each patient. The model emphasizes the dynamic interplay among determinants and supports operationalization through computational tools such as Bayesian Networks and rule-based scoring systems.

**Figure 2:**
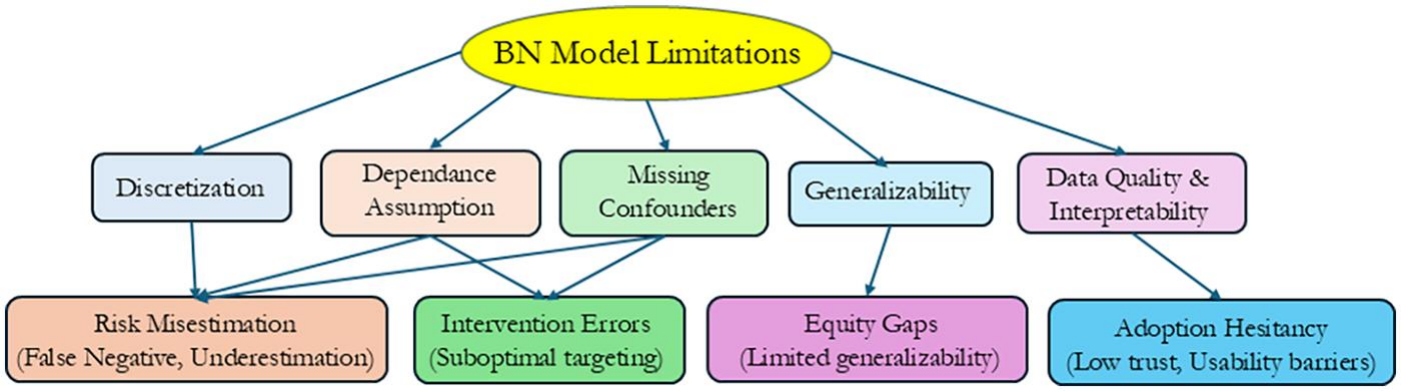
Obesity-relevant categories and sub-categories within the biology dimension of the PHDm. This diagram illustrates a subset of the hierarchical taxonomy applied to the obesity case study (see Section 3). Within the Biology dimension, the categories of Nutrition and Metabolic Biology are highlighted, along with selected sub-categories (e.g., Dietary Fiber under Nutrition and Insulin Sensitivity under Metabolic Biology). The figure demonstrates how the comprehensive PHDm taxonomy (theoretical maximum −72,000 elements) is selectively focused on clinically relevant factors for practical application. Full hierarchical lists for all nine dimensions are provided in Supplementary Tables S1–S3.

Building on the rationale and structure outlined above, the PHDm organizes each of its nine dimensions into a hierarchical taxonomy consisting of categories (up to 20 per dimension), sub-categories (up to 20 per category), and specific measurable health elements (up to 20 per sub-category). This yields a theoretical maximum of approximately 72,000 health elements, while in practice only a tailored, clinically relevant subset is applied to each patient. The complete hierarchical taxonomy is provided in Supplementary Tables S1–S3. These were derived from systematic synthesis of the frameworks above plus disease-specific literature. Figures 1, 2 illustrate the high-level structure and an obesity-relevant subset (Biology dimension), respectively; only clinically relevant subsets are used in practice.

For the obesity case study presented in this paper, we focus primarily on two dimensions, Biological and Lifestyle, to demonstrate how the model can be operationalized. Within the Biological dimension, we highlight the categories of Nutrition and Metabolic Biology. Within the Lifestyle dimension, we highlight Caloric Intake and Activity Frequency (see Table 1 and Supplementary Tables S1–S3 for the full taxonomy).

**Table 1:** Structure of PHDm showing the progression from the dimension to health condition for cases considered as an example in this study. Letters and numbers are codes distinguishing each element in each level.

Dimension (2/9)	Category (2/20) for each Dimension	Sub-Category (2/20) for each Category	Health Condition (1/20) for each Sub-Category
A.Biology	A.1.Nutrition	A.1.3.Dietary Fiber	A.1.3.8.Obesity
	A.17.Metabolic Biology	A.17.1.Insulin Sensitivity	A.17.1.5.Obesity
K.Lifestyle	K.1.Diet	K.1.3.Caloric Intake	K.1.3.1.Obesity
	K.2.Physical Activity	K.2.1.Activity Frequency	K.2.1.1.Obesity

Operationalizing this complexity requires advanced analytic methods. Accordingly, the PHDm integrates AI methods with established probabilistic and statistical modeling approaches, such as Bayesian Network Frameworks (BNFs) (34) and Linear Scoring Models (LSMs) (35) to pinpoint the root causes of various diseases. BNFs capture probabilistic dependencies among variables (e.g., linking sedentary behavior to obesity risk), while LSMs assign interpretable weights to selected factors to generate personalized risk scores. By focusing on condition-relevant subsets, these methods balance comprehensiveness with usability, offering healthcare professionals a scalable, adaptive framework for precision assessment and intervention.

### 3.1 Biology dimension

Biological factors are a central dimension of the PHDm, capturing genetic, molecular, and physiological influences on health. These determinants shape obesity risk through pathways spanning energy metabolism, fat storage, appetite regulation, and systemic inflammation. For example, impaired insulin signaling promotes visceral fat accumulation, while altered mitochondrial activity reduces fatty acid oxidation, both mechanisms strongly linked to obesity (19–21). Nutrition is also a critical driver: imbalances in macronutrient intake or insufficient dietary fiber consistently promote excess fat storage and metabolic dysfunction (36). Such biological processes form the foundation upon which psychological, social, and lifestyle factors act (e.g., interacting with the Lifestyle dimension via caloric intake), making them essential for precision obesity care.

Within the PHDm, biological determinants are systematically organized into categories and sub-categories (Table 1 and Supplementary Table S1). Figure 2 illustrates obesity-related categories and their related sub-categories for the Biology dimension. For this case study, two categories are emphasized: Nutrition and Metabolic Biology. Each is subdivided into measurable factors that can be operationalized within Bayesian Network Frameworks (BNFs) for risk modeling (Table 1 and Supplementary Table S2). Two illustrative sub-categories are highlighted: Dietary Fiber (from Nutrition) and Insulin Sensitivity (from Metabolic Biology).

The following examples are drawn from the scientific literature to demonstrate the type of data that can be integrated into the PHDm. They are not intended as an exhaustive review of biology or nutrition, but rather as representative cases showing how biological determinants can be quantified and translated into actionable, personalized strategies through AI-driven analysis.

#### 3.1.1 Dietary fiber

Dietary fiber (DF) is a critical factor within the Nutrition sub-category of the PHDm, with major implications for obesity management. Fiber enhances satiety, stabilizes blood glucose, and shapes gut microbiota in ways that reduce fat accumulation. High-fiber foods such as whole grains, fruits, vegetables, and legumes promote fullness by adding bulk and slowing digestion, thereby lowering overall caloric intake. Soluble fibers such as beta-glucans and pectin delay carbohydrate and fat absorption, improving insulin sensitivity and reducing fat storage. In addition, fermentation of fiber by gut microbiota produces short-chain fatty acids (SCFAs), which enhance fat oxidation and reduce obesity-related inflammation (37).

Clinical evidence supports these mechanisms. For example, higher daily fiber intake is consistently associated with lower body weight, reduced visceral fat, and improved metabolic health. Fiber-rich whole-food diets providing ≥30 g/day promote weight loss, reduce waist circumference, and improve lipid and glucose regulation (38). Daily supplementation with viscous fibers (such as PGX or psyllium) has also produced modest weight loss and significant reductions in waist circumference over 12 months (39). Across studies, increasing dietary fiber intake, whether from whole foods or supplements, reduces energy intake and produces meaningful weight loss (≈1.9 kg on average), with even greater effects in obese individuals. These findings support fiber intake above current U.S. levels as a strategy to help prevent obesity (40).

Within the PHDm, dietary fiber functions as a measurable biological determinant of obesity risk. By integrating fiber-related metrics into AI-driven models, the PHDm can help identify patients most likely to benefit from fiber-focused dietary interventions, supporting personalized and scalable obesity management strategies.

##### 3.1.1.1 Quantifying the impact of dietary fiber on obesity

The following metrics are selected examples from the literature, intended to illustrate how dietary fiber influences obesity. They are not exhaustive, but serve as representative inputs that can be mapped into the PHDm framework and incorporated into Bayesian Network (BN) or rule-based models.

##### 3.1.1.2 Body composition and energy balance

These outcomes can be discretized into thresholds (e.g., daily intake ≥25 g; waist circumference reduction ≥2 cm) for integration into BN models (e.g., as parent nodes to obesity risk edges). Some key findings are listed below:

- *Weight and BMI:* Consumption of an additional 14 g/day of fiber for more than 2 days is associated with a 10% decrease in energy intake and an average body weight loss of 1.9 kg over 3.8 months (40). Prospective studies show that for each 1 g of fiber per 1,000 kcal increase, body weight and body fat decrease by 0.25 kg and 0.25%, respectively (38). EPIC cohort data indicate that each 10 g/day increase in fiber intake is associated with a 39 g/year reduction in weight and a 0.08 cm/year reduction in waist circumference. Furthermore, each 8 g/day increment in fiber intake has been linked to an approximate 0.25 kg/m^2^ reduction in BMI (38). For each 10 g/day higher total dietary fiber intake, the pooled estimate for annual weight change was −39 g/year, corresponding to a BMI change of approximately −0.013 units in the study population (mean height = 1.69 m) (41).
- *Visceral Fat and Waist Circumference:* For every 10 g increase in soluble fiber, the rate of visceral adipose tissue (VAT) accumulation decreased by 3.7% (*P* = 0.01) (42).

##### 3.1.1.3 Metabolic regulation

These markers serve as intermediate biological variables, linking fiber intake to both energy balance and long-term disease risk in probabilistic models. Some key findings are listed below.

- *Glucose and Insulin:* In large cohorts like the Nurses' Health Study, fiber intakes ≥15 g/day significantly reduce type 2 diabetes risk (43).
- *Glucose and Insulin:* Specific fibers, such as 5 g/day of oat beta-glucan, lower postprandial glucose and insulin responses, underscoring fiber's role in glycemic control (43).
- *Lipid and Hormonal Effects: Fiber*-rich diets improve lipid profiles, reduce triglycerides, and lower hunger hormones, thereby curbing overeating (44).

##### 3.1.1.4 Gut Microbiota and inflammation

These emerging pathways broaden the model beyond traditional metabolic markers, incorporating microbiome and inflammation as additional causal nodes. Some key findings are listed below.

- *SCFA Production:* Fiber fermentation increases short-chain fatty acid production by ∼25%, enhancing fat oxidation and lowering visceral adiposity (45).
- *Microbiota Diversity and Inflammation:* High-fiber diets increase Bifidobacterium abundance and reduce C-reactive protein (CRP) by 15%–25%, mitigating obesity-related inflammation (46, 47).
- High-fiber diets, whether naturally rich in fiber or supplemented with fiber, can reduce C-reactive protein (CRP) levels by 14%–18%, indicating a modest reduction in inflammation. However, the effect is more pronounced in lean individuals compared to obese individuals, who did not experience significant reductions in CRP levels (48).

**Synthesis:** Evidence indicates that higher intake of dietary fiber, whether from whole-food sources or soluble/viscous fiber supplements, is modestly but consistently associated with improved body composition, reduced adiposity, and better metabolic regulation. For example, older adult data show that increasing fiber intake by 10 g/day was associated with minimal annual weight gain (∼ –39 g/year), which translates into only a small change in BMI (∼ –0.013 units/year). Some intervention meta-analyses of viscous fiber report modest but significant reductions in body weight, BMI and other adiposity parameters independent of calorie restriction.

Beyond weight and fat mass, higher fiber intakes, especially of soluble fiber, are associated with reduced accumulation of visceral adipose tissue (VAT). In one longitudinal study in youths, lower fiber intake was linked to substantial VAT increases over time, while higher fiber intake was linked to attenuation or reduction of visceral fat accumulation. More generally, fiber-rich diets have been linked to improved glycemic control, lower insulin resistance, and more favorable lipid profiles, including reductions in LDL-cholesterol and triglycerides.

Mechanistically, emerging evidence suggests that part of fiber's benefit may come from its effects on the gut microbiota, increasing microbial fermentation, production of short, chain fatty acids (SCFAs), and improvements in inflammation and metabolic signaling. Taken together, these findings support modeling fiber intake (e.g., ≥25–30 g/day) as a protective factor against obesity, excess visceral fat, and metabolic dysregulation, even if the magnitude of the effects on body weight or BMI remains modest and likely accumulative over time.

#### 3.1.2 Insulin sensitivity

Insulin sensitivity (IS) regulates glucose and lipid metabolism, making it a key determinant of obesity risk. High sensitivity allows efficient glucose uptake and limits fat storage, while insulin resistance promotes hyperinsulinemia, lipogenesis, and visceral fat accumulation (49).

Lifestyle and nutritional interventions can substantially improve insulin sensitivity. Supervised exercise interventions moderately improve insulin sensitivity in healthy adults (effect sizes: 0.38 post-intervention, 0.43 pre–post), reducing fasting insulin to 6.8 vs. 7.9 mU/L in controls—equivalent to a 30%–50% gain (50). Within the PHDm, insulin sensitivity is treated as a measurable metabolic factor that can be integrated with genetic, dietary, and behavioral data (e.g., as conditional probabilities in BN structures). Applied through AI-driven tools, these insights enable personalized risk assessment and targeted strategies to mitigate obesity and its complications.

##### 3.1.2.1 Quantifying the impact of insulin sensitivity

The following representative metrics are selected examples from the literature, intended to illustrate how insulin sensitivity influences obesity. They are measurable inputs that can be mapped into the PHDm framework and incorporated into BN or rule-based models.

##### 3.1.2.2 Body composition

Th following measures link body composition directly to insulin dynamics and can be encoded into BN models using thresholds for weight change or waist circumference (e.g., as parent nodes to obesity risk edges). Some key findings are:

- *Weight and BMI:* A 5% weight loss improves insulin sensitivity by −15%, while a 5% regain reduces it by −17% (51).
- *Body Fat:* Exercise-induced weight loss of −6.5% leads to a −32% increase in insulin sensitivity (52).
- *Waist Circumference:* A 32% improvement in insulin sensitivity was observed in the exercise weight loss group likely due to significant reductions in total and abdominal fat, emphasizing the critical role of abdominal adiposity in metabolic health (52).

##### 3.1.2.4 Metabolic and hormonal markers

These markers serve as intermediate variables that can be modeled as continuous or discretized states within BNs, capturing causal links between lifestyle changes and metabolic health. Some key findings are as follows:

- HOMA-IR and Ectopic Fat: An −9% body weight reduction decreases visceral fat by 32%, pancreatic fat by 42%, and liver fat by 84%, with HOMA-IR improving by −40% (53).
- Fasting Insulin: Intermittent fasting reduces fasting insulin by up to 50% while lowering BMI by −6% (54). Alternate-day fasting resulted in a mean weight loss of 6.0% after 12 months, which was comparable to the 5.3% weight loss observed in the daily calorie restriction group (55).
- Adiponectin: Caloric restriction of 20%–40% significantly increases adiponectin, improving insulin sensitivity and fat oxidation (56).

##### 3.1.2.5 Energy balance

These pathways connect insulin regulation to both energy expenditure and appetite, highlighting dynamic feedback loops suitable for BN modeling.

- Weight Maintenance & Insulin Levels: Individuals maintaining ≥15% weight loss for over 12 months show significantly lower fasting and 2 h insulin levels compared to controls (51).
- Appetite Regulation: Obesity leads to fat accumulation in adipose tissue, particularly visceral fat, which is associated with increased inflammation, ectopic fat deposition in insulin-sensitive tissues, and lipotoxicity (57).

**Synthesis:** Higher insulin sensitivity is strongly linked to weight loss, fat reduction (especially visceral/ectopic fat), and exercise. In one randomized controlled trial of middle-aged women, a −6.5% weight loss led to reductions in total, abdominal, and visceral fat — and only the group combining exercise + weight loss experienced a significant improvement in insulin sensitivity. Similarly, a 48-month intervention showed that weight loss initially produced large improvements in HOMA-IR (an insulin-resistance index), although the effect tended to wane if weight was regained.

Reductions in visceral or ectopic fat (e.g., liver fat) appear particularly important: a study of moderate weight loss in obese individuals found that decreases in liver fat strongly predicted improvements in insulin resistance, more so than reductions in visceral fat alone. Other work suggests that improvements in insulin sensitivity depend less on exercise-induced fat oxidation *per se*, and more on a reduction in circulating free fatty acid mobilization and uptake, which lowers lipotoxic stress on insulin-sensitive tissues.

Importantly, the effect size of insulin sensitivity improvements tends to scale with the magnitude and sustainability of weight/fat loss: small to moderate weight loss (≥3%–5%) can already yield measurable IS gains, especially when combined with exercise, but long-term maintenance of weight loss is critical to preserve those gains.

### 3.2 Lifestyle dimension

Lifestyle factors are a critical dimension of the PHDm, profoundly shaping obesity risk by influencing energy balance, metabolism, and behavior. Sedentary behavior reduces energy expenditure and increases obesity risk by 20%–30% (28). Dietary quality is also pivotal: in one U.S. cohort, nearly 40% of children's calories came from highly processed foods. A 10% increase in calories from ultra-processed foods was associated with a 2-point decrease in Healthy Eating Index (HEI-2015) scores, while a similar increase from minimally processed foods raised HEI scores by 3 points (29).

By contrast, lifestyle improvements have measurable benefits. Each additional 30 min per week of aerobic exercise reduces body weight by −0.5 kg, waist circumference by −0.6 cm, and body fat percentage by −0.4% (30). Sleep disruption, conversely, promotes excess snacking on calorie-dense foods, undermining weight loss and weight-maintenance efforts (31).

Within the PHDm, lifestyle determinants are systematically mapped and prioritized using AI-driven tools such as Bayesian Network Frameworks (BNFs). These tools enable personalized interventions that balance comprehensiveness with clinical feasibility. For the purposes of this case study, the Lifestyle dimension is represented by two sub-categories, Caloric Intake and Activity Frequency.

#### 3.2.1 Caloric intake

Diet is a primary determinant of obesity risk through its influence on energy balance, nutrient composition, and metabolic regulation. The global rise in obesity has been strongly linked to increased caloric intake, driven by the widespread availability of energy-dense, highly palatable foods. Diets rich in refined carbohydrates, added sugars, and ultra-processed products promote hyperphagia and a sustained positive energy balance by triggering insulin spikes, lowering satiety, and encouraging excessive consumption. U.S. adults derive well over 15% of their daily energy from added sugars, well above the <10% limit recommended by the FDA, with nearly half of this intake coming from sugar-sweetened beverages (32). These effects are further amplified by sedentary lifestyles, as elevated insulin levels promote fat storage and persistent hunger (58).

Conversely, diets emphasizing nutrient-dense and satiating foods—such as dietary fiber, lean proteins, and unsaturated fats—enhance satiety, improve insulin sensitivity, and support healthy metabolism. The Mediterranean diet, characterized by vegetables, fruits, legumes, whole grains, olive oil, and seafood, has been shown to improve metabolic profiles and facilitate weight loss when paired with caloric restriction. Similarly, plant-based diets promote satiety, reduce saturated fat intake, and improve inflammatory and lipid markers.

##### 3.2.1.1 Quantifying the impact of caloric intake

The following examples from the literature illustrate how caloric intake affects obesity through body composition, metabolism, and long-term risk. They are not exhaustive but demonstrate representative variables for integration into BN or rule-based models.

##### 3.2.1.2 Body composition metrics

These outcomes can be discretized into thresholds for BN modeling (e.g., as parent nodes to obesity risk edges). Some key findings are:

- Weight & BMI: The “3,500 kcal per pound” rule posits that a 500 kcal daily energy surplus or deficit produces roughly 0.5 kg of weekly weight change (59). While this rule oversimplifies the complex and dynamic processes underlying energy balance (60), it is employed in this model as a practical approximation.
- Body Fat: A 40% caloric surplus (≈950 kcal/day) over 8 weeks increased fat mass by −3.5 kg and lean mass by up to 3.2 kg, depending on protein intake (61).
- Waist Circumference: A 300 kcal/day deficit over 12 weeks reduced waist circumference by −2 cm and body weight by −3.5 kg in overweight individuals (62).

##### 3.2.1.3 Energy balance and metabolism

These findings highlight metabolic adaptations that must be captured in BN frameworks to avoid oversimplifying energy balance. Some key findings are:

- *Energy Content of Weight Loss:* The CALERIE I trial showed that the energy density of weight loss increases over time, from −4,800 kcal/kg at week 4 to −6,600 kcal/kg at week 24, reflecting shifts in body composition during caloric restriction (63).
- *Resting Metabolic Rate (RMR):* Overeating raises RMR through thermogenesis, but 60%–75% of excess calories are still stored as body fat (64).
- *Fat Oxidation:* Reduced-carbohydrate diets increase fat oxidation (−460 kcal/day higher) compared with reduced-fat diets, though both achieve weight loss (65).

##### 3.2.1.4 Metabolic & hormonal markers

These markers show how caloric intake interacts with hormonal regulation and inflammation, key pathways in BN models. Some key findings are:

- *Insulin Sensitivity:* In Hispanic women with prior GDM, higher calorie intake (>2,100 kcal/day) predicted a faster decline in insulin sensitivity and *β*-cell function (66).
- *Leptin Resistance:* Caloric surpluses elevate leptin, but resistance blunts satiety signaling, lowering thermogenesis and perpetuating weight gain (58).
- *Inflammation (CRP):* The study found that obese adolescents consumed significantly more calories (−500 kcal/day) than controls and had higher high-sensitivity C-reactive protein (hs-CRP) levels, indicating increased inflammation. However, no correlation was observed between hs-CRP levels and specific dietary constituents, including macronutrients, fiber, sugars, or adherence to the Mediterranean diet (67).

##### 3.2.1.5 Obesity risk & clinical evidence

These results confirm caloric intake as a central, modifiable determinant of obesity risk and intervention success:

- *Long-term Risk:* A study finds that very small deviations from energy balance, on the order of 1%–2% of daily energy intake, can result in large long-term changes in body weight (−20 kg). It emphasizes that when energy intake exceeds expenditure, the excess energy is deposited as body tissue, leading to weight gain (68).
- *RCT Evidence:* Intensive lifestyle interventions based on caloric restriction produce substantial and sustained weight loss. In the DPP, participants lost −7 kg at 6 months and maintained this at 12 months, outperforming pharmacologic groups (69).
- *RCT Evidence:* The Look AHEAD trial reported −8.6% weight loss at 12 months vs. <1% in controls (69).

**Synthesis:** Caloric intake is a central, modifiable driver of obesity risk because even small, sustained energy surpluses accumulate into significant long-term weight gain. Diets high in refined carbohydrates, added sugars, and ultra-processed foods promote excessive caloric intake by lowering satiety, triggering insulin spikes, and encouraging overeating, while sedentary lifestyles magnify these effects by favoring fat storage. Evidence shows that relatively modest positive energy balances—on the order of 500 kcal/day—can produce noticeable weekly weight gain, and even tiny chronic deviations (1%–2% of daily intake) can lead to −20 kg of weight change over time. Controlled studies demonstrate that caloric surpluses predominantly increase fat mass, whereas caloric restriction lowers body weight and waist circumference, with metabolic adaptations (such as shifts in resting metabolic rate and fat oxidation) shaping the rate and composition of weight change.

Hormonal and metabolic responses further reinforce the role of caloric burden: higher intakes impair insulin sensitivity, elevate leptin yet promote leptin resistance, and increase inflammatory markers such as CRP. Conversely, diets emphasizing fiber, lean protein, and unsaturated fats enhance satiety, improve insulin signaling, and reduce overall energy intake. Across randomized trials, interventions centered on caloric restriction consistently yield meaningful and sustained weight loss, outperforming pharmacological approaches. Collectively, these findings show that caloric intake influences body composition, metabolic function, and long-term obesity risk through tightly interconnected energy-balance and hormonal pathways.

#### 3.2.2 Activity frequency

The World Health Organization (33) guidelines, reaffirmed in a 2024 fact sheet, recommend that adults engage in at least 150–300 min of moderate-intensity aerobic physical activity or 75–150 min of vigorous-intensity aerobic physical activity per week, or an equivalent combination, along with muscle-strengthening activities involving all major muscle groups on 2 or more days a week (33). These recommendations underscore the value of consistent physical activity (PA) for reducing body fat and managing weight, among other benefits. For instance, a 2024 meta-analysis of 116 randomized trials involving over 6,800 adults with overweight or obesity found that aerobic exercise totaling at least 150 min per week (typically delivered in 3–5 sessions) over 6–12 months yielded an average 2.79 kg (approximately 3%–4% body weight) loss, with greater effects (up to 4.19 kg or −5%) at 300 min per week, alongside significant reductions in waist circumference and body fat percentage (30).

Within the PHDm, activity frequency is modeled as a measurable lifestyle factor (e.g., as probabilistic edges in BN structures) that can be operationalized in BNFs through thresholds.

##### 3.2.2.1 Quantifying the impact of activity frequency

The followings are illustrative examples from the literature showing how physical activity frequency impacts obesity, and represent measurable outcomes for BN integration.

##### 3.2.2.2 Body composition

Physical activity (PA) thresholds (e.g., ≥150 min/week moderate-to-vigorous physical activity (MVPA), Waist Circumference (WC) reduction ≥2.5 cm) can be encoded as conditional nodes in BN models.

- Weight & BMI: Increasing moderate-to-vigorous physical activity (MVPA) by ≥150 min/week led to −6.4 kg greater weight loss and −4.8 kg more fat loss at 18 months (70).
- Weight & BMI: Every 30 min/week increase in MVPA added −3.5 kg of additional weight loss (70).
- Body Fat %: Structured MVPA (≥150 min/week in ≥10 min bouts) significantly reduced fat mass (−4.8 kg more loss vs. < 150 min/week) (70).
- Waist Circumference: Reallocating 30 min to MVPA reduced waist circumference by −2.5 cm, compared with smaller effects from replacing sedentary time or sleep (71).

##### 3.2.2.3 Energy expenditure & Fat oxidation

These metrics link PA frequency to energy dynamics, suitable for BN modeling as quantitative levers:

- *Total Energy Expenditure (TEE):* ≥150 min/week of moderate or 75 min/week of vigorous aerobic activity (−1,000 kcal/wk) for health benefits, and ≥225 min/week (−2,000 kcal/wk) for weight loss or maintenance (72).
- *Fat Oxidation:* Maximal fat oxidation rate increased over 12 months of training (occurring at 35 ± 6% VO₂max pre-training, 44 ± 15% at 6 months, and 50 ± 14% VO₂max at 12 months, *P* < 0.01) (73).
- *Fat Oxidation:* MVPA was positively correlated with fat oxidation efficiency (*β* = 0.27, *p* = 0.04) (74).

##### 3.2.2.4 Metabolic & hormonal markers

These markers capture PA's regulatory effects, modeled as intermediate variables in BNs:

- *Insulin* Sensitivity: Each additional hour/day of MVPA increased QUICKI index scores (75).
- *Insulin Sensitivity:* Programs with ≥170 min/week improved sensitivity by −80%–90%, compared to −40% with shorter sessions (76).
- *Leptin:* Children with obesity who engaged in MVPA had significantly lower leptin levels than peers who were sedentary or engaged in only light activity (77).

##### 3.2.2.5 Obesity risk & clinical outcomes

These outcomes highlight PA frequency's role in prevention and maintenance, as causal nodes in BN structures:

- *Weight* Maintenance: After bariatric surgery, individuals with low PA were 3.25 times more likely to regain >5% of lost weight than those with moderate PA (78).
- *Long-term Weight Loss*: Participants who maintained ≥10% weight loss at 6 and 24 months reported −275 min/week of leisure-time PA, equivalent to −1,800 kcal/week (79).
- *Obesity Incidence:* Increasing MVPA by ≥150 min/week during supervised programs reduced obesity risk and was associated with −6.4 kg greater weight loss at 18 months (70).

**Synthesis:** Regular physical activity has a clear, dose-dependent effect on obesity risk and weight regulation. Guidelines reaffirmed by the WHO in 2024 recommend 150–300 min of moderate or 75–150 min of vigorous activity per week, and extensive evidence shows that meeting or exceeding these thresholds produces meaningful reductions in body weight, fat mass, and waist circumference. Aerobic exercise at ≥150 min per week typically yields 3%–4% weight loss over 6–12 months, with larger benefits approaching 5% at 300 min per week. More frequent or longer bouts of moderate-to-vigorous activity amplify these effects: each 30-minute increase in weekly MVPA can add −3.5 kg of additional long-term weight loss, and total reductions in fat mass and waist circumference scale proportionally with training volume. These relationships reflect both increased total energy expenditure and improved fat oxidation capacity over time.

Beyond weight change, higher activity frequency produces powerful metabolic and hormonal improvements that further reduce obesity risk (80). Programs providing ≥150–170 min of MVPA per week substantially enhance insulin sensitivity (up to 80%–90% improvement in some studies), lower leptin levels, and shift substrate use toward greater fat oxidation. Consistency also appears crucial for weight-regain prevention: individuals with low activity are over three times more likely to regain significant weight after bariatric surgery, whereas long-term weight-loss maintainers typically sustain −275 min of weekly activity. Overall, the evidence shows that frequent, sustained physical activity is one of the strongest lifestyle predictors of lower obesity risk, improved metabolic health, and durable weight control. Significant enhancements in both fine and gross motor skills following selected exercise interventions in Iraqi children aged 8–9 years, is reported with gender playing a moderating role (81). These findings further support the inclusion of activity frequency as a key actionable element within the Lifestyle dimension of the PHDm, particularly for pediatric populations.

The following section demonstrates how these quantified lifestyle and biological determinants can be integrated within a Bayesian Network to model obesity risk and infer personalized intervention pathways.

## 4 Bayesian network for an obesity model

This section demonstrates the application of the PHDm within a BN framework to estimate obesity risk. The BN approach supports precision health by quantifying individualized risk profiles and identifying potential intervention pathways.

To illustrate this process, four representative factors are modeled: Dietary Fiber (DF), Insulin Sensitivity (IS), Caloric Intake (CI), and Activity Frequency (AF). These are drawn from the Nutrition, Metabolic Biology, Diet, and Physical Activity categories within the Biology and Lifestyle dimensions of the PHDm (Table 1). Focusing on this subset highlights how the model can translate complex, multidimensional data into clinically feasible and computationally tractable analyses.

### 4.1 Overview of Bayesian networks

**Figure 3:**
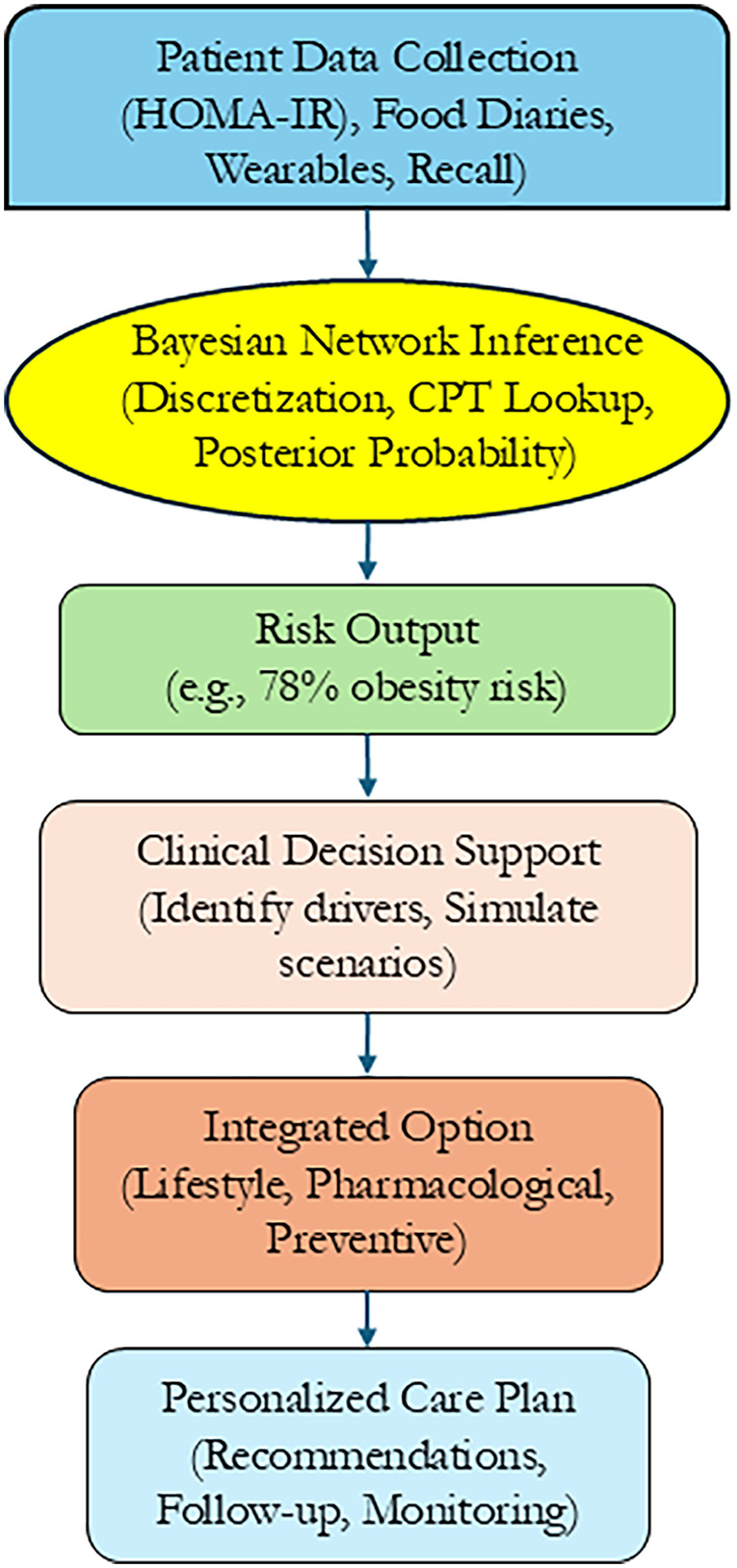
Simplified Bayesian network structure for obesity risk within the PHDm (direct-effect model). This figure illustrates the core structure used in the illustrative operationalization (Section 4). The four exemplar factors—Dietary Fiber (DF), Insulin Sensitivity (IS), Caloric Intake (CI), and Activity Frequency (AF)—are modeled as parent nodes with direct edges to the target node Obesity (O). Marginal prior probabilities and selected conditional probabilities are shown for clarity. This computationally tractable structure serves as a proof-of-concept demonstration of how the PHDm can translate multidimensional health determinants into probabilistic risk assessment.

A Bayesian Network (BN) is a probabilistic graphical model that represents variables as nodes and their conditional dependencies as directed edges within a directed acyclic graph (DAG). In the obesity model, DF, IS, CI, and AF are modeled as parent nodes that directly influence the target node, obesity (O). The edges represent conditional dependencies supported by clinical evidence (Figure 3).

**Figure 4:**
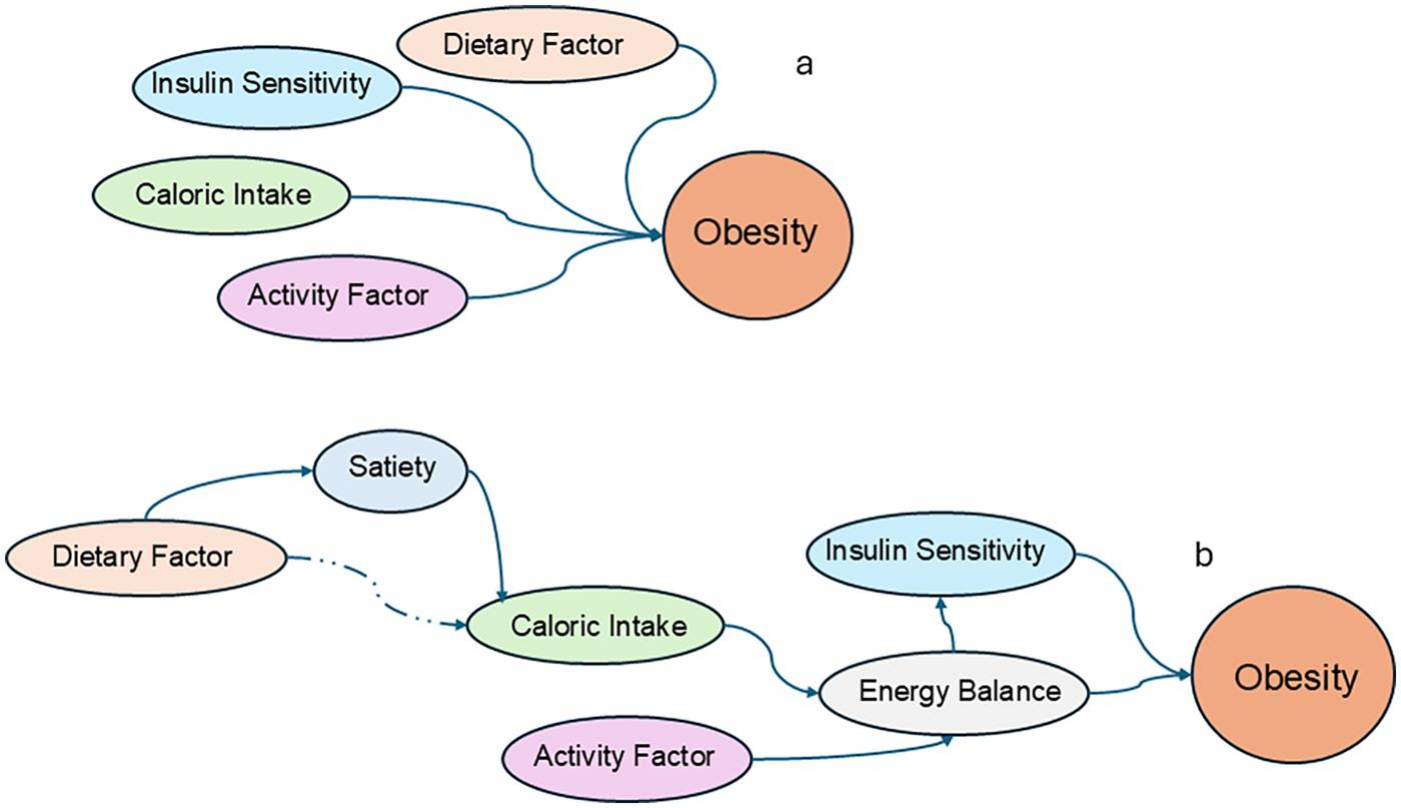
Two illustrative Bayesian network structures for obesity risk in the PHDm. **(a)** Simplified direct-effect model in which Dietary Fiber, Insulin Sensitivity, Caloric Intake, and Activity Frequency exert direct influences on Obesity. This version assumes conditional independence among parent nodes for computational simplicity. **(b)** Physiologically informed model incorporating indirect pathways derived from the literature synthesis in Sections 2.0 and 3. Dietary Fiber influences Caloric Intake via Satiety; Activity Frequency affects Energy Balance; and Insulin Sensitivity interacts with Energy Balance. Dashed lines indicate plausible but context-dependent relationships. These structures demonstrate how expert knowledge grounded in clinical evidence can be operationalized within the PHDm framework.

The structures in Figure 4 were constructed using expert knowledge informed by the mechanistic evidence synthesized in Sections 2.0 and 3. For clarity, the current DAG in Figure 4a represents a simplified structure in which the four exemplar factors (Dietary Fiber, Insulin Sensitivity, Caloric Intake, and Activity Frequency) exert direct effects on obesity. This structure assumes conditional independence among the parent nodes for computational tractability and ease of interpretation. Figure 4b illustrates a more physiologically plausible structure incorporating indirect pathways supported by the scientific literature reviewed in Sections 2.0 and 3. Specifically, Dietary Fiber influences obesity via Satiety and subsequent effects on Caloric Intake; Activity Frequency influences obesity via Energy Balance; and Insulin Sensitivity interacts with Energy Balance. These edges were determined through expert knowledge elicitation informed by the evidence synthesis presented in Sections 2.0 (rationale and literature review) and 3 (detailed quantification of each factor's mechanisms). No automated structure-learning algorithm (e.g., PC algorithm or score-based methods) was applied in this illustrative case study; instead, the structure reflects established causal relationships documented in the clinical and epidemiological literature (e.g., fiber → satiety → caloric intake; physical activity → energy balance → obesity risk).

This hybrid approach—expert-driven structure with literature-grounded edges—balances physiological fidelity with model simplicity. It directly demonstrates how the detailed evidence review in Sections 2.0 and 3 informs the PHDm's operationalization. Future iterations of the model will incorporate data-driven structure learning (e.g., using constraint-based or hybrid algorithms on NHANES or similar cohorts) to refine or validate these expert-derived structures.

### 4.2 Selected variables and states

To balance computational efficiency with clinical relevance, continuous variables were discretized into binary states using evidence-based thresholds (Table 2):

- *Dietary Fiber (DF):* Adequate (≥ 28 g/day, based on USDA recommendations) = 0; Low (< 28 g/day) = 1 (82).
- *Insulin Sensitivity (IS):* High (HOMA-IR < 2.5) = 0; Resistant (HOMA-IR ≥ 2.5) = 1 (83).
- *Caloric Intake (CI):* Balanced (within ± 100 kcal of daily requirements) = 0; Excessive (≥500 kcal/day surplus) = 1 (68).
- *Activity Frequency (AF):* Frequent (≥3 sessions/week, −150 min of moderate activity) = 0; Infrequent (< 3 sessions/week) = 1 (33).
- *Obesity (O):* Non-obese (BMI < 30 kg/m^2^) = 0; Obese (BMI ≥ 30 kg/m^2^) = 1 (84).

**Table 2:** Evidence-based thresholds of factors affecting obesity.

Node	Favorable State (0)	Unfavorable State (1)	Source
Dietary Fiber (DF)	Adequate (≥28 g/day)	Low (<28 g/day)	(82)
Insulin Sensitivity (IS)	High (HOMA-IR <2.5)	Resistant (HOMA-IR ≥2.5)	(83)
Caloric Intake (CI)	Balanced (within ±100 kcal/day of requirements)	Excessive (≥500 kcal/day surplus)	(68)
Activity Frequency (AF)	Frequent (≥3 sessions/week, −150 min moderate activity)	Infrequent (<3 sessions/week)	(33)
Obesity (O)	Non-obese (BMI <30 kg/m^2^)	Obese (BMI ≥30 kg/m^2^)	(84)

These thresholds align with widely accepted clinical guidelines and epidemiological data, providing a practical yet simplified framework for personalized health assessments. These binary thresholds were selected for computational simplicity and clinical interpretability in this illustrative prototype. However, optimal cutoffs may vary by age, sex, ethnicity, metabolic status, and geographic region (e.g., dietary fiber recommendations differ across populations, and HOMA-IR cutoffs show ethnic variability). Future implementations will incorporate continuous or multi-state variables and population-specific calibration. Sensitivity analyses on threshold values were performed and are reported in Section 4.6 and the Supplementary Material.

Obesity was defined in this prototype as BMI ≥ 30 kg/m^2^ (O = 1) for consistency with major epidemiological datasets (e.g., NHANES) and to enable straightforward binary classification. We acknowledge that BMI has well-known limitations as a sole indicator of obesity-related risk. In precision health applications, it should be complemented by measures such as waist circumference, body fat percentage, visceral adipose tissue (VAT) volume, and metabolic obesity phenotypes (e.g., metabolically unhealthy normal-weight individuals). Ethnicity-specific BMI cutoffs (e.g., ≥27.5 kg/m^2^ for some Asian populations) are also clinically relevant. Future extensions of the PHDm will incorporate multi-dimensional obesity outcomes and continuous risk scores.

### 4.3 Marginal and conditional probabilities

Marginal probabilities describe the likelihood of a single variable's state. For instance, P(IS = 1) = 0.27 indicates that approximately 27% of the global adult population has insulin resistance. While marginal probabilities were anchored in NHANES and epidemiological data, most conditional probabilities were derived from literature-based odds ratios and subsequently calibrated (see Section 4.6 for details).

Conditional probabilities capture the likelihood of obesity given the states of its parent variables, expressed as P(O = 1 | IS, DF, CI, AF). For example, P(O = 1 | IS = 1, DF = 1, CI = 1, AF = 1) ≈ 0.75, indicating a 75% risk of obesity when all risk factors are present, while P(O = 1 | IS = 0, DF = 0, CI = 0, AF = 0) ≈ 0.15 illustrates protection in the absence of risk factors. These values are derived from clinical studies on BMI, visceral fat, and insulin resistance, with interpolations applied where data gaps exist using multiplicative odds ratios (OR) effects for synergy (e.g., joint OR ≈ product of singles, adjusted via logistic: log(p/(1-p)) = *β*_0_ + *Σ β*_i_ * state_i_) (See more details in Supplementary Material, Bayesian Network Structure section).

#### 4.3.1 Example conditional probability tables (CPTs)

To operationalize the BN, each node requires a Conditional Probability Table (CPT) specifying P(node | parents). For parent nodes (DF, IS, CI, AF), CPTs are marginal priors (Table 3) based on population distributions (e.g., from NHANES). For the child node O, the CPT assumes conditional independence given parents for simplicity, with probabilities derived from meta-analytic odds ratios (ORs) converted to probabilities (assuming a base P(O = 1) ≈ 0.403 from recent US data (84, 85).

**Table 3:** Example of marginal priors for parents.

Node	Abb.	P(State = 0) Favorable	P(State = 1) Unfavorable	Source
Dietary Fiber	DF	0.45	0.55	Approx. US intake distribution
Insulin Sensitivity	IS	0.73	0.27	IS prevalence in adults
Caloric Intake	CI	0.70	0.30	Surplus prevalence estimates
Activity Frequency	AF	0.69	0.31	Insufficient activity rates

#### 4.3.2 Simplified CPT for obesity (assuming independence; single-parent examples)

For brevity, Table 4 lists marginal conditionals for each parent. Derived from ORs: low DF OR≈1.35 (83), IR OR≈2.0 (86), excess CI OR≈1.8 (68), low AF OR≈1.25 (33).

**Table 4:** Simplified CPT table for obesity listing marginal conditionals for each parent.

Parents	State		P(O = 0)	P(O = 1)
Dietary Fiber	Adequate	(DF = 0)	0.65	0.35
	Low	(DF = 1)	0.55	0.45
Insulin Sensitivity	High	IS = 0	0.70	0.30
	Resistant	IS = 1	0.40	0.60
Caloric Intake	Balanced	CI = 0	0.65	0.35
	Excessive	CI = 01	0.45	0.55
Activity Frequency	Frequent	AF = 0	0.65	0.35
	Infrequent	AF = 1	0.55	0.45

In practice, joint P(O = 1 | all parents) could be computed via d-separation in BN software, yielding personalized posteriors (e.g., P(O = 1 | DF = 1,IS = 1,CI = 1,AF = 1) ≈ 0.75 under multiplicative ORs). This enables “what-if” simulations for interventions, such as shifting AF from 1 to 0 to reduce risk by −20%. The full 16-state table for all combinations would multiply OR effects, e.g., via logistic approximation (See the section on Logistic Regression Parameterization in Supplementary Material).

### 4.4 Constructing the Bayesian network

The DAG structure, informed by expert consensus and published evidence, includes directed edges from IS, DF, CI, and AF to O (Figure 4a), reflecting their established roles as obesity risk factors (87, 88). Marginal probabilities for parent nodes (e.g., P(IS = 1) = 0.27 (Table 3), corresponding to the global prevalence of insulin resistance in adult populations) were derived from epidemiological datasets. Conditional Probability Tables (CPTs) for O (Table 4) were then populated using effect sizes from clinical studies, with odds ratios (ORs) converted via logistic approximation to capture how combinations of dietary, biological, and lifestyle factors shape obesity risk. Table 5 summarizes key ORs for unfavorable states, supporting the edges and synergies (e.g., multiplicative for joint effects).

**Table 5:** Suggested odds ratio (Ors) for risk factors in the Bayesian network (BN).

Factor	Suggested OR (Unfavorable State)	Rationale & Citation	Why It Fits the BN
Low Dietary Fiber (DF = 1)	≈1.35 (low fiber vs. adequate)	NHANES 1999–2018 meta: Q4 (≥20.8 g/day) vs. Q1 (≤9.1 g/day) OR = 0.74 for obesity; inverse implies 1.35 (74). Alternative: RR 0.77 for high fiber (27).	Ties to fiber metrics (≥28 g/day threshold); dose-response aids discretization.
Excessive Caloric Intake (CI = 1)	≈1.8 (surplus ≥500 kcal/day)	Prospective cohorts: Dynamic modeling (60).	Aligns with surplus threshold; supports “what-if” simulations.
Low Activity Frequency (AF = 1)	≈1.25 (infrequent vs. frequent)	Cohort meta: Insufficient PA OR1.25–1.5 for incident obesity (62).	Matches ≥150 min/week; frequency fits PHDm activity.
Insulin Resistance or Sensitivity state IR (IS = 1)	≈2.0 (resistant vs. sensitive)	Meta: IR in obese OR2.0 for T2DM/obesity progression (77).	Links to HOMA-IR ≥2.5; causal for edges.

This framework operationalizes the PHDm into a probabilistic system capable of individualized risk estimation and scenario testing, calibrated via NHANES for initial validation.

#### 4.4.1 Conditional probability table (CPT)

Table 6 presents a partial CPT for the Obesity node, exemplifying selected combinations of the four binary parent variables (IS, DF, CI, AF) out of the full 16 states. Probabilities were estimated from clinical evidence and adjusted to reflect synergistic effects; for example, insulin resistance combined with caloric excess amplifies obesity risk beyond additive effects (e.g., via joint OR ≈3.0). Where direct data were unavailable, interpolated values were applied using established epidemiological trends and logistic conversion of ORs (e.g., OR≈1.35 for low DF; OR≈1.8 for excess CI; OR≈1.25 for low AF; OR≈2.0 for IR). The full CPT in Supplementary Material Table S4 provides the foundation for Bayesian Network-based inference, enabling individualized obesity risk estimation and prioritization of interventions, such as increasing dietary fiber or physical activity.

**Table 6:** Partial CPT for the obesity node, using selected combinations of the four binary parent variables insulin sensitivity(iS), dietary fiber (DF), caloric intake (CI), and activity frequency (AF) out of the full 16 states.

IS	DF	CI	AF	P(O = 0)	P(O = 1)
0	0	0	0	0.85	0.15
1	0	0	0	0.40	0.60
0	1	0	0	0.55	0.45
0	0	1	0	0.45	0.55
0	0	0	1	0.55	0.45
1	1	1	1	0.25	0.75

### 4.5 Examples of conditional probabilities

The following conditional probabilities, derived from clinical studies, illustrate how the PHDm's Bayesian Network captures obesity risk under different scenarios:

- Insulin Resistance: P(O = 1 | IS = 1) ≈ 0.60. Individuals with insulin resistance (HOMA-IR ≥ 2.5) have a 3%–5% increase in body fat and 10%–15% higher visceral fat, elevating obesity risk (89, 90).
- Low Dietary Fiber: P(O = 1 | DF = 1) ≈ 0.45. Fiber intake below 28 g/day is associated with higher obesity risk, whereas a 14 g/day increase reduces body weight by −1.9 kg over 3.8 months (91).
- Caloric Excess + Low Activity: P(O = 1 | CI = 1, AF = 1) ≈ 0.65. A 500 kcal/day surplus combined with <150 min/week of activity results in weight gains of 0.5–1 kg per month (59).
- Combined IS + DF Deficits: P(O = 1 | IS = 1, DF = 1) ≈ 0.70. Insulin resistance paired with low fiber intake, common in metabolic syndrome, strongly increases obesity risk (92, 93).
- Protective States: P(O = 1 | IS = 0, DF = 0, CI = 0, AF = 0) ≈ 0.15. High insulin sensitivity, adequate fiber, balanced intake, and frequent activity together minimize obesity risk (93).

These examples illustrate how conditional probabilities translate clinical evidence into quantifiable risk estimates. Within the PHDm, such probabilities enable Bayesian inference to identify high-risk profiles and highlight intervention priorities, such as increasing fiber intake or physical activity frequency. Single factors such as insulin resistance or low dietary fiber elevate risk, but combinations yield synergies akin to metabolic syndrome, while favorable states underscore the power of holistic patterns. By quantifying these dynamics, the PHDm structures intervention prioritization and clinical scenario simulations.

### 4.6 Detailed Bayesian network methodology

This section consolidates the methodological steps used to construct, parameterize, and evaluate the Bayesian Network (BN) prototype within the PHDm framework.

#### 4.6.1 Data sources

Marginal probabilities and model calibration were primarily informed by the National Health and Nutrition Examination Survey (NHANES) subset (*n* = 10,000 participants with available BMI and relevant proxy variables) (94). Conditional probabilities and odds ratios were derived from published meta-analyses, systematic reviews, and large cohort studies (see Tables 4–6 and Supplementary Table S4). Full details of proxy variable mapping are provided in the Supplementary Material.

#### 4.6.2 Variable coding and discretization

Continuous variables were discretized into binary states using evidence-based thresholds (Table 2):

- *Dietary Fiber (DF):* Adequate (≥28 g/day) = 0; Low (<28 g/day) = 1
- *Insulin Sensitivity (IS):* High (HOMA-IR < 2.5) = 0; Resistant (HOMA-IR ≥ 2.5) = 1
- *Caloric Intake (CI):* Balanced (within ±100 kcal/day) = 0; Excessive (≥500 kcal/day surplus) = 1
- *Activity Frequency (AF):* Frequent (≥150 min/week) = 0; Infrequent (<3 sessions/week) = 1
- *Obesity (O):* Non-obese (BMI <30 kg/m^2^) = 0; Obese (BMI ≥ 30 kg/m^2^) = 1

These thresholds were chosen for clinical interpretability and computational tractability in this illustrative prototype.

#### 4.6.3 Structure definition

The directed acyclic graph (Figure 4) was developed using expert knowledge elicitation informed by the mechanistic evidence synthesized in Sections 2.0 and 3. Figure 4a uses a simplified direct-effect structure for computational tractability. Figure 4b incorporates key indirect pathways supported by clinical literature. No automated structure-learning algorithms were applied in this illustrative demonstration.

#### 4.6.4 CPT construction and parameter estimation

Conditional Probability Tables (CPTs) for the Obesity node were constructed using a hybrid approach:

- Marginal (prior) probabilities for the four parent nodes were derived from NHANES and global epidemiological data (Table 3) (85).
- Conditional probabilities were primarily elicited from published meta-analyses and odds ratios using logistic approximation, with multiplicative synergy terms for biologically plausible interactions (e.g., insulin resistance + low dietary fiber) (86, 87). Full CPTs are provided in Supplementary Table S4.
- Calibration on NHANES: Maximum Likelihood Estimation (MLE) via the *pgmpy* library (95) was applied to the NHANES subset to refine marginal probabilities and validate overall model fit.

#### 4.6.5 Validation procedure, performance metrics, and sensitivity analysis

Predictive performance was assessed using 10-fold cross-validation on the NHANES subset. Using a 0.5 probability threshold for binary obesity classification (O = 1), the Bayesian Network achieved approximately 85% accuracy (precision 0.82, recall 0.80) (96). The rule-based scoring system showed −80% concordance with BN outputs on the same dataset.

Sensitivity analyses were conducted by varying key thresholds (e.g., dietary fiber 25–30 g/day, HOMA-IR 2.0–3.0, caloric surplus 300–700 kcal/day). Results showed stable model rankings and risk classifications across reasonable ranges, although absolute probabilities varied modestly. These analyses confirm that while the prototype thresholds are convenient for demonstration, population-specific calibration will be essential for broader application.

All analyses were conducted in Python using the *pgmpy* library. Detailed variable mappings, proxy definitions, code snippets, and the full CPTs are available in the Supplementary Material. Full code and datasets will be shared upon publication via a public repository.

##### 4.6.5.1 Illustrative examples

- All unfavorable states (IS = 1, DF = 1, CI = 1, AF = 1): P(O = 1 | E) ≈ 0.75 (Table 6).
- All favorable states (IS = 0, DF = 0, CI = 0, AF = 0): P(O = 1 | E) ≈ 0.15 (Table 6).
- Partial evidence (IS = 1, CI = 1, AF = 1; DF unknown): Inference yields P(O = 1 | E) ≈ 0.728 (see Table 7 for marginalization details).

A summary comparison of the BN and rule-based approaches is provided in Table 8.

**Table 7:** Marginalization using marginal P(DF) and conditional P(DF | E).

Marginalization Using Marginal P(DF)			
State	P(O = 1 | IS = 1, DF, CI = 1, AF = 1)	P(DF)	Contribution
DF = 0	0.70	0.45	0.70 × 0.45 = 0.315
DF = 1	0.75	0.55	0.75 × 0.55 = 0.413
Total | P(O = 1 | E) **=** 0.315 + 0.413 = 0.728			

**Marginalization Using Conditional P(DF | E)**			
State	P(O = 1 | IS = 1, DF, CI = 1, AF = 1)	P(DF | E)	Contribution
DF = 0	0.70	0.50	0.7 × 0.5 = 0.350
DF = 1	0.75	0.50	0.75 × 0.5 = 0.375
Total | P(O = 1 | E) = 0.350 + 0.375 = 0.725			

IS, insulin sensitivity; DF, dietary fiber; CI, caloric intake; AF, activity frequency; E, stands for evidence.

**Table 8:** summary of methodological approach and data sources for the PHDm obesity model.

Aspect	Bayesian Network (BN)	Rule-Based Scoring System
Purpose	Probabilistic risk estimation and “what-if” intervention simulation	Rapid, transparent risk stratification
Structure Definition	Expert knowledge + literature synthesis (Sections 2.0 & 3)	Same four factors as BN
Parameterization	Hybrid: Literature-derived ORs + logistic approximation; marginals calibrated on NHANES	Weights derived from BN CPTs and literature effect sizes
Data Sources	Literature/meta-analyses for CPTs; NHANES (n = 10,000) for marginals, calibration, and validation (proxies)	Same as BN
Training/Calibration	Maximum Likelihood Estimation (MLE) via pgmpy on NHANES subset	Not applicable (deterministic)
Validation	10-fold cross-validation on NHANES subset; sensitivity analyses on thresholds	Concordance with BN outputs (−80%) on same dataset
Performance	−85% accuracy (precision 0.82, recall 0.80)	−80% concordance with BN
Software/Implementation	Python (pgmpy library)	Manual or spreadsheet
Key Strengths	Handles uncertainty, missing data, dependencies, and scenario simulation	High interpretability, no software required
Limitations	Computationally heavier; relies on proxies	Slightly lower granularity
Best Use Case	Data-rich clinical/research settings	Low-resource/point-of-care settings

Both approaches use the same four PHDm factors and evidence-based thresholds. Full technical details (variable proxies, CPTs, code snippets, and sensitivity analyses) are provided in the Supplementary Material.

### 4.7 Illustrative clinical application and workflow

The following section describes a hypothetical clinical workflow to illustrate how the Personalized Health Determinants Model (PHDm) could be applied once fully developed, validated, and integrated into clinical systems. Importantly, the current implementation is a proof-of-concept prototype based on only four simplified obesity-related factors (Insulin Sensitivity, Dietary Fiber, Caloric Intake, and Activity Frequency) and binary thresholds. It has not undergone prospective clinical validation, regulatory review, or real-world testing and should not be used as a clinical decision-support tool at this stage.

#### 4.7.1 Proposed workflow (illustrative only)

**Figure 5:**
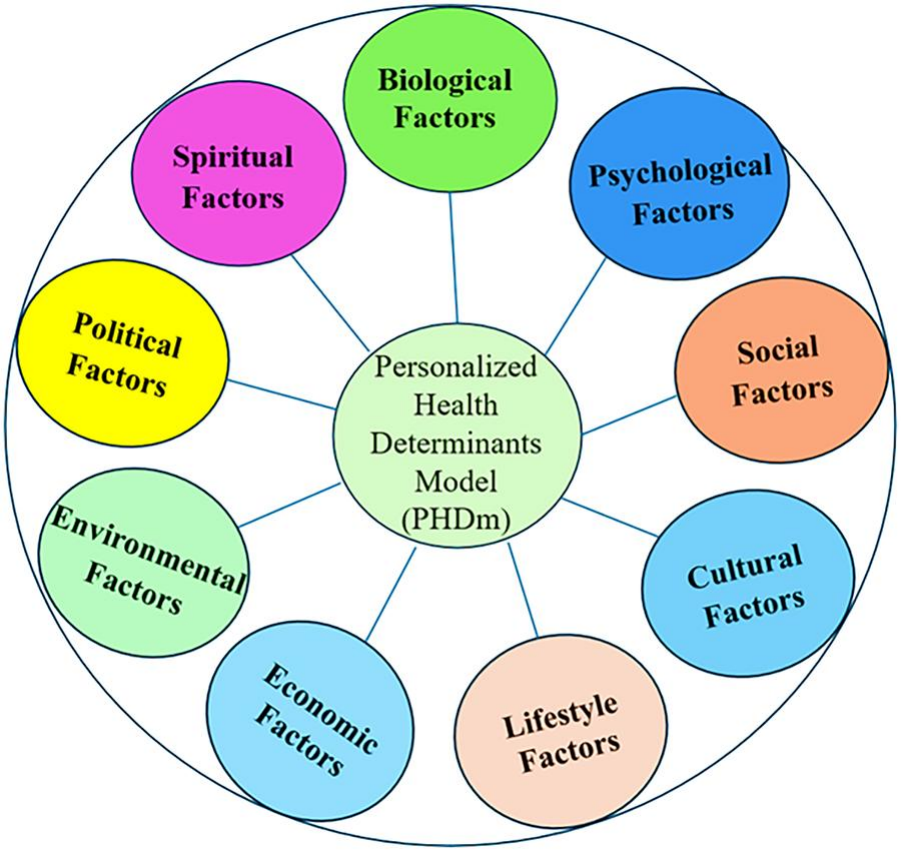
Clinical workflow for personalized obesity risk assessment and intervention planning using the PHDm. The process begins with patient data collection (clinical measures, dietary logs, wearables), followed by input into the Bayesian Network or rule-based scoring system. Posterior risk probabilities are computed, key risk drivers are identified, and “what-if” intervention scenarios are simulated. The output supports personalized care planning, including lifestyle, pharmacological, or combined strategies. This workflow illustrates the practical translation of the PHDm from conceptual framework to clinical application.

The workflow, summarized in Figure 5, consists of the following steps:

(i) *Risk Assessment:* Patient-specific data are collected from multiple sources, including electronic health records (e.g., HOMA-IR for insulin sensitivity), dietary recalls or food frequency questionnaires (for dietary fiber and caloric intake), and wearable devices or self-reports (for activity frequency). These data are discretized using evidence-based thresholds and entered into either the Bayesian Network or the rule-based scoring system. For example: A 45-year-old patient presents with insulin resistance (IS = 1) and low dietary fiber intake (DF = 1). The prototype BN returns a posterior probability of obesity P(O = 1) ≈ 0.70, while the rule-based score classifies the patient as High Risk (5+ points).
(ii) *Identification of Key Drivers and Intervention Simulation:* The model highlights the strongest contributing factors and enables “what-if” scenario testing. Example: Increasing dietary fiber to adequate levels (DF = 0) while maintaining other factors is simulated, reducing the estimated risk from 0.70 to approximately 0.60. Similarly, improving activity frequency can be modeled to quantify potential risk reduction.
(iii) *Personalized Care Planning:* Based on the prototype risk estimate and driver analysis, clinicians could consider tailored recommendations such as:
  (a) Dietary counseling focused on fiber-rich foods
  (b) Structured physical activity programs (e.g., ≥150 min/week moderate activity)
  (c) Pharmacological support when clinically indicated (e.g., metformin for insulin resistance)
  (d) Referral to multidisciplinary teams for broader PHDm dimensions (psychological, social, economic, etc.)
(iv) *Monitoring and Iteration:* Follow-up measurements would allow iterative updating of the model as new data become available.

#### 4.7.2 Critical caveats

This workflow is presented strictly for illustrative purposes. The current PHDm prototype uses a highly simplified subset of factors and binary discretization to demonstrate feasibility and interpretability. Many important determinants, continuous relationships, interactions, and patient-specific contextual factors are not yet incorporated. The model is not clinically validated and should not be deployed for patient care. Prospective clinical studies, external validation in diverse populations, integration with electronic health records, and regulatory approval will be necessary before any real-world clinical application.

This illustrative example is intended to stimulate discussion, refinement, and collaboration toward the eventual development of robust, equitable precision health tools.

## 5 Scenario analysis

- The model enables “what-if” simulations.
- Example: A patient with all unfavorable states (IS = 1, DF = 1, CI = 1, AF = 1) has P(O = 1) ≈ 0.75. Improving DF and AF (to DF = 0, AF = 0) yields P(O = 1) ≈ 0.60, a 20% risk reduction. Summary of further examples are provided in Table 9.

**Table 9:** further clinical examples.

Example	Profile	Model Output (Rationale)	Application & Plan
1. Middle-aged patient with partial data	45-year-old, IS = 1 (HOMA-IR = 3.0), CI = 1 (+600 kcal/day), AF = 1 (< 3 sessions/week), DF unknown.	P(O = 1) ≈ 0.73 (via marginalization over DF).	Prioritize caloric restriction and exercise; assess fiber at follow-up. Plan: Refer to dietitian for 500 kcal/day deficit; prescribe 150 min/week brisk walking (52).
2. Young adult with metabolic risk	30-year-old, IS = 1, DF = 1, CI = 0, AF = 0.	P(O = 1) ≈ 0.65 (combined IS + DF deficits).	Address insulin resistance and low fiber. Plan: Initiate metformin; recommend +14 g/day fiber (27).
3. Preventive care for low-risk patient	25-year-old, IS = 0, DF = 0, CI = 0, AF = 0.	P(O = 1) ≈ 0.15 (protective states).	Focus on maintenance. Plan: Counselling on diet/activity; annual monitoring.

Risk Categories.

• 0–2 points: Low Risk [P(O = 1) ≤25%].

• 3–4 points: Moderate Risk [P(O = 1) 26%–50%].

• 5–7 points: High Risk [P(O = 1) >50%].

Weights are based on BN CPTs and effect sizes. The system achieves −80% concordance with the Bayesian Network on NHANES data.

### 5.1 Implementation considerations

The Bayesian Network (BN) within the PHDm framework supports real-time obesity risk estimation by fusing heterogeneous data streams—dietary logs, activity-tracker metrics, and clinical indicators such as HOMA-IR—into a unified probabilistic model. Conditional Probability Tables (CPTs) can be iteratively updated via AI-assisted parameter learning, with optimization performed through cross-validation on large datasets (e.g., NHANES) using established BN toolkits such as *bnlearn* (97) Table 10.

**Table 10:** Rule-Based scoring weights and risk categories for obesity risk.

Factor/Combination	Unfavorable State	Weight	Source
Insulin Sensitivity (IS)	HOMA-IR ≥ 2.5	3	(89)
Dietary Fiber (DF)	<28 g/day	1	(88)
Caloric Intake (CI)	≥500 kcal/day surplus	1	(60)
Activity Frequency (AF)	<150 min/week	1	(70)
Synergy (IS + DF)	Both IS = 1 and DF = 1	+1	(87)

The current BN relies on binary states and direct dependencies, which improves interpretability but restricts modeling of nonlinear, multiscale metabolic interactions. Future versions could incorporate continuous variables, hierarchical state representations, indirect pathways, and fuzzy-logic formalisms to better accommodate uncertainty, gradation, and digitization noise in behavioral and physiological inputs. Fuzzy membership functions, for example, could operationalize constructs such as satiety or adherence, while machine-learning–based structure learning (e.g., PC algorithm extensions) enhances physiological fidelity and model adaptability.

### 5.2 Benefits

- *Personalization:* Tailored to drivers (e.g., fiber for DF = 1).
- *Proactive Management:* Flag high-risk [P(O = 1) >0.70] for prevention.
- *Evidence-Based:* Derived from clinical data (60, 98).
- *Flexibility:* Handles incomplete data; suits resource-constrained settings.

### 5.3 Challenges

- *Data Accuracy:* Self-reported CI/AF unreliable; prefer objective measures.
- *Model Limitations:* Binary discretization (e.g., HOMA-IR ≥2.5) omits confounders (e.g., genetics).
- *Adoption Barriers:* Clinician unfamiliarity and time constraints.
- *Equity Concerns:* Western calibration; validate with global cohorts for generalizability.

Together, these considerations highlight the potential of the PHDm to transform obesity management through AI-driven precision health, while underscoring the importance of validation, clinician training, and equity-focused adaptation before large-scale implementation.

## 6 Limitations

**Figure 6:**
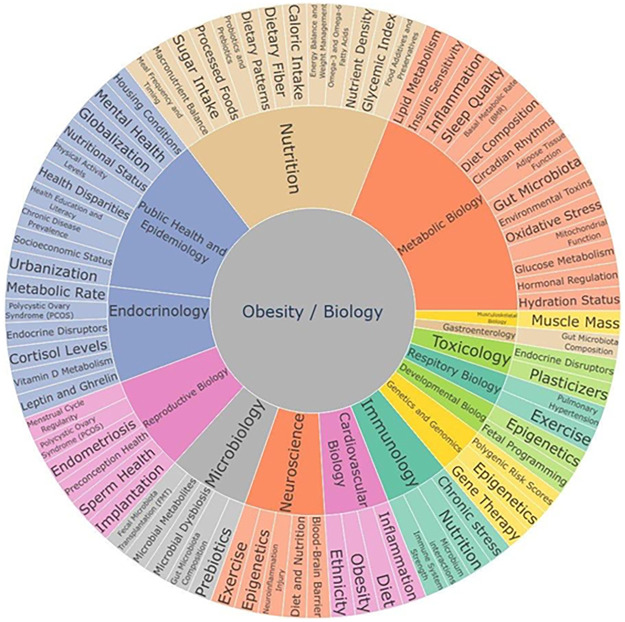
Clinical implications of key limitations of the Bayesian network implementation within the PHDm. The figure summarizes how discretization, missing confounders, dependence assumptions, and generalizability issues may translate into clinical risks (e.g., risk misestimation, suboptimal intervention targeting, equity gaps, and adoption barriers). Mitigation strategies and directions for future refinement are highlighted. As a proof-of-concept demonstration, these limitations underscore the need for further validation and model enhancement in diverse populations.

While the PHDm's BN model offers a tractable framework for obesity risk estimation, several limitations must be addressed to enhance precision, equity, and clinical utility. These are outlined below, with mitigations for future refinement (Figure 6). Future studies will perform fully data-driven parameter and structure learning on cohorts with richer dietary and biomarker data.

As a proof-of-concept demonstration, the current implementation relies on only four simplified obesity-related factors and binary thresholds. This design choice prioritizes interpretability and tractability for illustration purposes but necessarily omits many important determinants and dose-response relationships. The PHDm in its present form is an illustrative prototype and is not yet ready for clinical deployment as a decision-support tool. Prospective validation in diverse real-world settings, integration with electronic health records, and regulatory considerations will be essential next steps.

### 6.1 Discretization

Continuous variables (e.g., HOMA-IR for IS, dietary fiber intake, caloric surplus) are binarized for computational efficiency and interpretability in this prototype. While convenient, these fixed thresholds may not generalize across age, sex, ethnicity, metabolic status, or geographic populations. For example, dietary fiber recommendations and insulin resistance cutoffs show notable variation internationally. This approach flattens dose-response relationships and risks misclassification near cutoff values (88).

**Mitigation:** Future iterations will explore multi-state (e.g., ternary) or continuous Bayesian Networks, fuzzy logic, or individualized thresholds. Sensitivity analyses on threshold selection were conducted and demonstrated reasonable robustness (see Section 4.6).

### 6.2 Missing confounders and dependence assumptions

The model omits key factors, e.g., FTO genetics (99); or sleep <6 h/night (100) or <6–7 h/night (101) and assumes parent independence (e.g., ignoring DF → CI via satiety), leading to incomplete profiles and biased marginalization (e.g., underestimating P(O = 1|IS = 1, DF = 1) if CI correlates with DF = 1).

#### 6.2.1 Mitigation

Incorporate high-impact variables via feature selection; add edges (e.g., DF → CI) or use data-driven learning (e.g., PC algorithm); validate with datasets similar to NHANES.

### 6.3 Generalizability

The current model was calibrated primarily on Western NHANES data (−42% obesity prevalence) and literature predominantly from high-income settings. Thresholds (e.g., DF ≥28 g/day, HOMA-IR ≥2.5) may not be optimal for non-Western or ethnically diverse populations, where baseline dietary fiber intake, body composition norms, and genetic predispositions differ substantially. This risks biased risk estimates and reduced accuracy in global contexts.

#### 6.3.1 Mitigation

Population-specific recalibration, use of relative rather than absolute thresholds, and multi-center validation using diverse datasets (e.g., WHO cohorts, regional biobanks) will be prioritized in future work to improve equity and transportability.

### 6.4 Additional pitfalls

- *Data Quality:* Self-reports (CI, AF) prone to bias; lab variability affects IS (e.g., recall errors up to 20%). Mitigation: Prioritize objective tools (e.g., wearables, CGMs); add uncertainty in inference.
- *Complexity vs. Usability:* Four-factor limit aids feasibility but sacrifices depth vs. ML alternatives. Mitigation: Feature selection for 6–8 factors; EHR dashboards for interpretability.
- *Interpretability:* Probabilistic outputs [e.g., P(O = 1) = 0.70] may confuse users. Mitigation: Visual aids (e.g., risk charts); clinician training on reasoning.

### 6.5 Implications for clinical Use

These limitations risk misestimation (e.g., false negatives from confounders), suboptimal targeting (e.g., ignoring sleep), inequity (e.g., Western bias), and low adoption (e.g., opacity). Yet, mitigations like modular designs and objective data can enhance value (Figure 6).

- *Risk Misestimation:* Use finer models/dependencies to stabilize P(O = 1).
- *Intervention Targeting:* Add causal pathways for holistic plans.
- *Equity/Adoption:* Global validation and user-friendly interfaces to build trust.

### 6.6 Scalability constraints

Limiting to four factors avoids CPT explosion (16 vs. 32 entries for five), but expansion (e.g., genetics, sleep) increases burden in resource-poor settings. Mitigation:

- *Short-term:* Prioritize via selection;
- *Medium-term:* Modular BNs (cap at 6–8 factors) or rule-based alternatives;
- *Long-term:* Automated pipelines (e.g., EHR-wearables).

### 6.7 Choice of obesity outcome measure (new subsection)

The current prototype defines the target node “Obesity (O)” using the conventional binary threshold of BMI ≥ 30 kg/m^2^. While this definition facilitates model training, validation against population data, and interpretability, it has important limitations for precision health. BMI does not distinguish between fat mass and lean mass, nor does it capture fat distribution (e.g., visceral vs. subcutaneous). It also fails to identify metabolically unhealthy normal-weight or metabolically healthy obese individuals (102).

More nuanced and clinically relevant measures include waist circumference (≥102 cm men/ ≥88 cm women), body fat percentage, visceral adipose tissue volume (via DXA or MRI), and composite metabolic obesity phenotypes. Additionally, ethnicity-specific BMI cutoffs are recommended by several guidelines (e.g., lower thresholds for Asian populations) (102).

Mitigation: Future iterations of the PHDm will expand the outcome layer to include multiple obesity-related endpoints (continuous or multi-class) and will support personalized risk profiling that integrates body composition, fat distribution, and metabolic health markers. Sensitivity analyses using alternative outcome definitions (e.g., waist circumference or metabolic syndrome) are planned.

## 7 Rule-based scoring example for obesity risk

A rule-based scoring system provides a straightforward, computationally lightweight alternative to the Bayesian Network for estimating obesity risk within the PHDm framework. By assigning weighted points to unfavorable states of the same four factors used in the BN (Insulin Sensitivity, Dietary Fiber, Caloric Intake, and Activity Frequency) and summing them, patients can be classified into Low, Moderate, or High risk categories. This system approximates the BN model's predictive performance while offering greater simplicity and interpretability.

**Justification for the rule-based approach**

The rule-based scoring system is justified on both practical and clinical grounds:

- Accessibility and usability: Many clinicians, especially in under-resourced environments, lack ready access to BN software or the time to run probabilistic inference. A simple additive score allows immediate risk stratification without digital tools.
- Interpretability: Unlike black-box machine learning models, the scoring system makes the contribution of each factor (and synergies) fully transparent to both clinicians and patients.
- Clinical performance: On a NHANES subsample, the rule-based system achieved 80% concordance with the BN model and comparable classification accuracy (80% overall), while maintaining strong alignment with literature-derived risk relationships.
- Complementary strengths: The two approaches together illustrate the flexibility of the PHDm, the BN for data-rich, precision-medicine environments and the rule-based system for rapid screening and low-infrastructure deployment. This dual presentation directly responds to calls for scalable, equitable tools in precision health.

The scoring system uses the same four factors (Insulin Sensitivity, Dietary Fiber, Caloric Intake, and Activity Frequency) and the same evidence-based thresholds as the BN (Section 4.2), ensuring consistency across methods. Weights were derived from the BN's conditional probabilities and literature effect sizes (Table 5), with a small synergy bonus for biologically plausible interactions (e.g., Insulin Resistance + Low Dietary Fiber).

### 7.1 Scoring methodology

Each unfavorable state is assigned points as follows:

- Insulin Sensitivity (IS = 1, resistant): 3 points
- Dietary Fiber (DF = 1, low): 1 point
- Caloric Intake (CI = 1, excessive): 1 point
- Activity Frequency (AF = 1, infrequent): 1 point
- Synergy bonus (IS = 1 + DF = 1): +1 point

#### 7.1.1 Risk classification

Total scores (range 0–7) are mapped to the following categories, calibrated against BN-derived probabilities:

- 0–2 points: Low Risk [P(O = 1) ≤25%]
- 3–4 points: Moderate Risk [P(O = 1) 26%–50%]
- 5–7 points: High Risk [P(O = 1) >50%]

### 7.2 Example applications

Example 1: Middle-Aged Patient with Insulin Resistance

- Profile: IS = 1 (HOMA-IR = 3.0), DF = 0, CI = 0, AF = 0
- Score: IS = 1 → 3 points
- Risk Category: Moderate Risk
- Corresponding BN probability: ≈0.60

Note: Although the BN indicates approximately 60% probability for insulin resistance alone, the simplified additive rule-based system classifies this scenario as Moderate Risk. In real-world settings, insulin resistance is rarely isolated and is typically accompanied by other unfavorable factors that elevate the total score into the High Risk category.

Example 2: Young Adult with Metabolic Risk

- Profile: IS = 1, DF = 1, CI = 0, AF = 0
- Score: IS = 1 (3 points) + DF = 1 (1 point) + synergy bonus (1 point) = 5 points
- Risk Category: High Risk (>50%)
- Corresponding BN probability: ≈0.70
- Plan: Initiate metformin (if clinically indicated); counsel increase of 14 g/day dietary fiber; reassess HOMA-IR in 3 months.

Example 3: Low-Risk Patient

- Profile: IS = 0, DF = 0, CI = 0, AF = 0
- Score: All favorable states → 0 points
- Risk Category: Low Risk (≤25%)
- Corresponding BN probability: ≈0.15
- Plan: Provide educational materials on nutrition and exercise maintenance; continue annual monitoring.

The performance and practical characteristics of the Bayesian Network and rule-based scoring approaches within the PHDm framework are compared in Table 11. The BN offers higher predictive accuracy (−85%) and superior handling of uncertainty and missing data, while the rule-based system provides speed, transparency, and usability in resource-limited settings.

**Table 11:** Comparison of Bayesian network and rule-based scoring approaches within the PHDm framework.

Aspect	Bayesian Network (BN)	Rule-Based Scoring System
Purpose	Probabilistic risk estimation and “what-if” intervention simulation	Rapid, transparent risk stratification
Computational Demand	Moderate (requires software: pgmpy)	Very low (manual or spreadsheet)
Interpretability	High (probabilistic)	Very high (explicit point weights and synergies)
Handling of Uncertainty	Excellent (marginalization over missing data)	Moderate (deterministic)
Key Strengths	Higher accuracy, captures dependencies and synergies	Accessibility in low-resource settings, ease of use
Performance	−85% accuracy (precision 0.82, recall 0.80) on NHANES	−80% concordance with BN; comparable classification accuracy
Risk Output	Continuous probability P(O = 1)	Categorical (Low/Moderate/High)
Best Clinical Use Case	Data-rich environments, research, detailed planning	Point-of-care, resource-limited settings, quick screening
Limitations	Requires software and training	Slightly lower granularity and precision

Both approaches are built on the same four PHDm factors (IS, DF, CI, AF) and evidence-based thresholds. The dual presentation demonstrates the flexibility and scalability of the PHDm for different resource contexts. Performance metrics are based on 10-fold cross-validation and concordance analysis on a NHANES subsample.

## 8 Discussion and conclusion

This manuscript introduces the Personalized Health Determinants Model (PHDm) as a comprehensive conceptual framework that expands Engel's Bio-Psycho-Social model into nine dimensions and demonstrates its illustrative operationalization through a Bayesian Network and a complementary rule-based scoring system applied to obesity risk. By integrating biological (Insulin Sensitivity) and lifestyle (Dietary Fiber, Caloric Intake, Activity Frequency) factors, the prototype shows how a tailored subset of the PHDm's theoretical −72,000 health elements can be translated into quantifiable risk estimates and intervention simulations.

The inclusion of both Bayesian Network and rule-based approaches highlights the PHDm's flexibility across different resource settings. Strengths of the current work include its structured synthesis of existing frameworks, transparent hybrid parameterization, and explicit demonstration of how multidimensional determinants can be operationalized. However, as a proof-of-concept prototype based on four simplified factors and binary thresholds, the model has important limitations (detailed in Section 6) and should not yet be viewed as a ready-to-deploy clinical tool.

### 8.1 Future directions

Substantial further work is required before the PHDm can meaningfully contribute to clinical precision health. Priorities include:

- Expansion to continuous variables, additional dimensions, and richer datasets;
- Fully data-driven structure and parameter learning;
- External validation across diverse populations, age groups, and geographic contexts;
- Prospective clinical studies and integration with electronic health records.

In conclusion, the PHDm provides a structured, hierarchical, and testable framework for integrating multiple health determinants in precision health. While the illustrative obesity case study demonstrates promising preliminary performance, rigorous empirical validation across diverse datasets and clinical contexts is essential. We invite collaboration among researchers, clinicians, AI developers, and funders to refine, validate, and scale this framework toward more equitable and personalized healthcare.

## Data Availability

The original contributions presented in the study are included in the article/Supplementary Material, further inquiries can be directed to the corresponding author/s.
