# Supplementary material for: From Engel's Bio-Psycho-Social model to the personalized health determinants model: a comprehensive framework and illustrative operationalization for precision health
Source: Front Digit Health. 2026 Jun 10;8:1763691. doi: 10.3389/fdgth.2026.1763691 (PMC13290953; doi:10.3389/fdgth.2026.1763691)
Supplement: Supplementary file 1 [file Datasheet1.pdf]

Supplementary Material

Table SM1. Main Categories and their Sub-categories considered in PHDm.

Dimension	Category
A.Biological	**Nutrition**, Microbiology, Immunology, Endocrinology, Genetics & Genomics, Pathology, Neuroscience, Cardiovascular Biology, Respiratory Biology, Reproductive Biology, Developmental Biology, Oncology, Hematology, Gastroenterology, Dermatology, Musculoskeletal Biology, **Metabolic Biology**, Toxicology, Infectious Diseases, Public Health, and Epidemiology.
B.Psychological	Stress Levels, Coping Mechanisms, Personality Traits, Mental Health Disorders, Self-Esteem, Social Support, Health Behaviors, Sleep Quality, Resilience, Cognitive Function, Emotional Regulation, Beliefs about Health, Life Satisfaction, Mindfulness, Adverse Childhood Experiences (ACEs), Social Isolation, Trauma and PTSD, Motivation, Attitudes toward Aging, Fear and Anxiety.
C.Social	Socioeconomic Status, Education, Employment, Social Support Networks, Access to Healthcare, Housing, Neighborhood and Built Environment, Food Security, Transportation, Cultural Norms, Racism and Discrimination, Social Exclusion, Safety and Security, Environmental Conditions, Political Climate, Economic Stability, Public Health Policies, Media and Technology, Legal Status, Early Life Conditions.
D.Cultural	Dietary Habits, Perceptions of Health and Illness, Language Barriers, Health Literacy, Traditional Healing Practices, Religious Beliefs, Gender Roles, Family Structure and Dynamics, Socioeconomic Factors, Stigma and Discrimination, Attitudes towards Aging, Body Image Norms, Workplace Cultures, Educational Attainment, Attitudes towards Disabilities, Cultural Competency of Healthcare Providers, Social Support Networks, Migration Patterns, Media Influence, Privacy and Confidentiality Concerns.
E.Environmental	Air Quality, Water Quality, Climate Change, Housing Conditions, Exposure to Chemicals, Radiation Exposure, Noise Pollution, Urbanization, Waste Disposal, Biodiversity, Land Use, Extreme Weather Events, Indoor Air Quality, Green Spaces, Transportation Options, Soil Quality, Microbial Contaminants, Electromagnetic Fields, Occupational Hazards, Lifestyle Interactions.
F.Economic	Income Level, Employment Status, Health Insurance Coverage, Housing Affordability, Food Security, Education Level, Social Services, Public Transportation, Economic Stability, Economic Inequality, Taxation Policies, Investment in Healthcare Infrastructure, Social Insurance Programs, Minimum Wage Levels, Inflation, Economic Policies, Debt Levels, Workplace Safety and Regulations, Retirement Savings and Pensions, Recession.
G.Political	Healthcare Policy and Legislation, Public Health Funding, Health Insurance Regulations, Environmental Regulations, Vaccination Policies, Food and Drug Regulations, Tobacco and Alcohol Policies, Education Policies, Social Welfare Policies, Housing Policies, Labor Laws and Workplace Safety, Income Inequality and Taxation Policies, Immigration Policies, War and Conflict Decisions, Climate Change Policies, Criminal Justice Policies, Gender Equality Policies, Corruption Levels, International Aid and Diplomacy, Emergency Preparedness Policies.
H.Spiritual	Sense of Purpose, Connectedness, Hope, Forgiveness, Gratitude, Faith in a Higher Power, Prayer/Meditation, Religious Practices, Compassion, Mindfulness, Spiritual Well-being, Ethical Living, Community Involvement, Moral Values, Acceptance of Death, Altruism, Spiritual Crisis, Inspirational Experiences, Sacred Texts, Transcendental Experiences.
K.Lifestyle	**Diet**, **Physical Activity,** Sleep, Smoking, Alcohol Consumption, Stress Management, Hydration, Sun Exposure, Body Weight, Personal Hygiene, Social Connections, Mental Health Care, Occupational Hazards, Recreational Drug Use, Screen Time, Environmental Quality, Safety Practices, Immunizations, Oral Health, Work-Life Balance.

Table SM2. Sub-Categories associated with Categories and Dimensions. Bold Sub-Categories are considered in this study.

Dimension	Category	Sub-Category
A.Biology	A.1.Nutrition	Macronutrient Balance, Micronutrient Intake, **Dietary Fiber**, Hydration, Caloric Intake, Antioxidants, Omega-3 and Omega-6 Fatty Acids, Probiotics and Prebiotics, Sugar Intake, Salt Intake, Processed Foods, Food Additives and Preservatives, Nutrient Density, Glycemic Index, Meal Frequency and Timing, Dietary Patterns, Food Allergies and Intolerances, Energy Balance and Weight Management, Nutrient Interactions, Supplement Use
	A.17.Metabolic Biology	**Insulin Sensitivity**, Basal Metabolic Rate (BMR), Hormonal Regulation, Nutrient Absorption, Mitochondrial Function, Lipid Metabolism, Glucose Metabolism, Protein Metabolism, Ketone Body Production, Adipose Tissue Function, Gut Microbiota, Inflammation, Oxidative Stress, Genetic Factors, Diet Composition, Physical Activity, Sleep Quality, Hydration Status, Circadian Rhythms, Environmental Toxins
K.Lifestake	K1.Diet	Macronutrients Balance, Micronutrient Intake, **Caloric Intake**, Hydration, Fiber Intake, Whole vs. Processed Foods, Sugar Consumption, Sodium Intake, Saturated and Trans Fats, Omega-3 Fatty Acids, Meal Timing and Frequency, Portion Control, Gut Microbiome Health, Food Quality, Allergies and Intolerances, Dietary Diversity, Alcohol Consumption, Supplement Use, Cooking Methods, Environmental Impact
	K.2.Physical Activity	**Activity Frequency,** Activity Intensity, Activity Duration, Type of Exercise, Muscle Strengthening, Cardiovascular Fitness, Flexibility and Mobility, Balance Training, Sedentary Time Reduction, Consistency, Recovery and Rest, Warm-Up and Cool-Down, Hydration During Exercise, Nutrition for Activity, Sleep Quality, Stress Levels, Body Posture and Technique, Environmental Conditions, Age-Appropriate Activities, Social and Emotional Support

Table SM3. Health issue associated with Sub-Category and Dimension. Obesity is considered as the health condition in an example.

Dimension	Sub-Category	Health issues
A.1.Nutrition	Dietary Fiber	Constipation, Irritable Bowel Syndrome (IBS), Diverticulosis/Diverticulitis, Colon Cancer, Hemorrhoids, Type 2 Diabetes, Hyperlipidemia, **Obesity**, Cardiovascular Disease, Gastroesophageal Reflux Disease (GERD), Gallstones, Inflammatory Bowel Disease (IBD), Metabolic Syndrome, Colorectal Adenomas, Diverticular Disease, Breast Cancer, Prostate Cancer, Chronic Kidney Disease, Pancreatic Cancer, Depression
A.17. Metabolic Biology	A.17.1.Insulin Sensitivity	Type 2 Diabetes, Metabolic Syndrome, Polycystic Ovary Syndrome (PCOS), Non-Alcoholic Fatty Liver Disease (NAFLD), **Obesity**, Hypertension, Cardiovascular Disease, Dyslipidemia, Prediabetes, Gestational Diabetes, Chronic Inflammation, Alzheimer's Disease, Sarcopenic Obesity, Osteoporosis, Hyperinsulinemia, Sleep Apnea, Cancer (e.g., Breast, Colon), Gout, Peripheral Neuropathy, Skin Disorders (e.g., Acanthosis Nigricans)
K1.Diet	Caloric Intake	**Obesity**, Underweight, Type 2 Diabetes, Cardiovascular Disease, Metabolic Syndrome, Hypertension, Non-Alcoholic Fatty Liver Disease, Malnutrition, Osteoporosis, Gout, Eating Disorders, Digestive Issues, Hyperlipidemia, Immune Dysfunction, Gallstones, Cancer Risk, Sleep Apnea, Hormonal Imbalance, Chronic Fatigue, Mental Health Disorders
K.2.Physical Activity	Activity Frequency	**Obesity**, Cardiovascular Disease, Type 2 Diabetes, Osteoporosis, Sarcopenia, Depression, Anxiety, Hypertension, Metabolic Syndrome, Arthritis, Low Back Pain, Sleep Disorders, Immune Dysfunction, Cognitive Decline, Coronary Artery Disease, Stroke, Chronic Fatigue Syndrome, Cancer Risk, Falls in Elderly

Supplementary Material

Bayesian Network Structure and Implementation Details

Overview

This supplementary material provides additional technical details for the Bayesian Network (BN) implementation described in Section 4 of the main manuscript. The BN models obesity risk within the Personalized Health Determinants Model (PHDm) using four exemplar factors from the Biology and Lifestyle dimensions: Dietary Fiber (DF), Insulin Sensitivity (IS), Caloric Intake (CI), and Activity Frequency (AF). These parent nodes influence the target node Obesity (O).

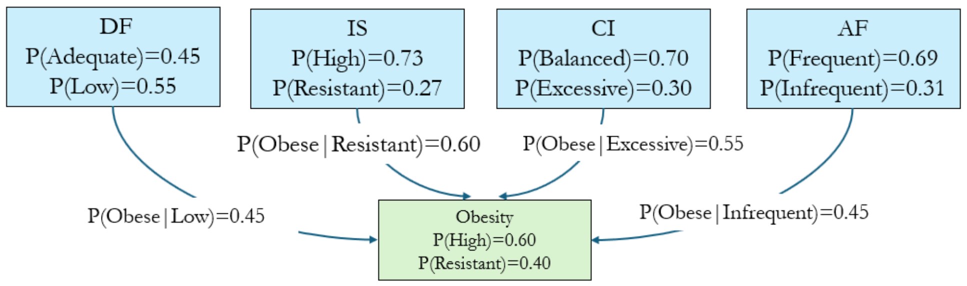

Nodes and States

All variables are discretized into binary states using evidence-based clinical thresholds (see main text Table 2 for details and rationale).

Structure Definition

The directed acyclic graph (DAG) was constructed using expert knowledge informed by the literature synthesis in Sections 2.0 and 3. Two structures are presented:

- A simplified direct-effect model (main text Figure 4a).
- A physiologically informed model with indirect pathways (main text Figure 4b).

No automated structure-learning algorithms were used in this illustrative prototype.

Marginal Priors

Marginal (prior) probabilities for the parent nodes are provided in main text Table 3.

Conditional Probability Table (CPT) for Obesity (O)

Conditional probabilities were constructed using a hybrid approach: literature-derived odds ratios converted via logistic approximation, with multiplicative synergy terms for biologically plausible interactions. The full 16-state CPT is provided in Table SM4 below.

Implementation Details

- *Software:* Python using the pgmpy library.
- *Parameter Estimation:* Hybrid (literature-based priors and conditional probabilities + Maximum Likelihood Estimation calibration on an NHANES subset).
- **Inference:** Variable Elimination.
- *Validation:* 10-fold cross-validation on NHANES data.
- *Code and Data:* Full implementation code, proxy variable mappings, and datasets will be made publicly available upon publication.

Sensitivity Analyses

- Sensitivity analyses were performed by varying key discretization thresholds (e.g., dietary fiber 25–30 g/day, HOMA-IR 2.0–3.0, caloric surplus 300–700 kcal/day). Model rankings and risk classifications remained stable across reasonable ranges.

Table SM4. Full Conditional Probability Table for Obesity (O)

Configuration	P(parents)	P(O=1 | parents)	P(O=0 | parents)	P(all, O=1)	P(all, O=0)
DF=0 IS=0 CI=0 AF=0	0.1586	0.1894	0.8297	0.0300	0.1286
DF=0 IS=1 CI=0 AF=0	0.0586	0.4392	0.5608	0.0257	0.0329
DF=1 IS=0 CI=0 AF=0	0.1939	0.2831	0.7169	0.0549	0.1390
DF=1 IS=1 CI=0 AF=0	0.0717	0.5696	0.4304	0.0408	0.0308
DF=0 IS=0 CI=1 AF=0	0.0679	0.3800	0.6200	0.0258	0.0421
DF=0 IS=1 CI=1 AF=0	0.0251	0.6726	0.3274	0.0169	0.0082
DF=1 IS=0 CI=1 AF=0	0.0831	0.5088	0.4912	0.0422	0.0408
DF=1 IS=1 CI=1 AF=0	0.0307	0.7764	0.2236	0.0238	0.0068
DF=0 IS=0 CI=0 AF=1	0.0712	0.2469	0.7531	0.0175	0.0536
DF=0 IS=1 CI=0 AF=1	0.0263	0.5235	0.4765	0.0138	0.0125
DF=1 IS=0 CI=0 AF=1	0.0871	0.3565	0.6435	0.0310	0.0560
DF=1 IS=1 CI=0 AF=1	0.0322	0.6500	0.3500	0.0209	0.0112
DF=0 IS=0 CI=1 AF=1	0.0306	0.4623	0.5377	0.0141	0.0164
DF=0 IS=1 CI=1 AF=1	0.0113	0.7424	0.2576	0.0083	0.0029
DF=1 IS=0 CI=1 AF=1	0.03734	0.5924	0.4076	0.0221	0.0152
DF=1 IS=1 CI=1 AF=1	0.01381	0.8297	0.1703	0.0114	0.0023
